# A reference material for X-ray diffraction line profile analysis

**DOI:** 10.1107/S1600576725006946

**Published:** 2025-09-18

**Authors:** P. Scardi, M. D’Incau, M. A. Malagutti, M. W. Terban, B. Hinrichsen, A. N. Fitch

**Affiliations:** a Department of Civil, Environmental and Mechanical Engineering University of Trento via Mesiano 77 38123Trento Italy; b Momentum Transfer GmbH, Luruper Hauptstraße 1, 22547Hamburg, Germany; c European Synchrotron Radiation Facility (ESRF) 71 Avenue des Martyrs 38000Grenoble France; Brazilian Synchrotron Light Laboratory, Brazil

**Keywords:** reference materials, steel, X-ray diffraction, whole powder pattern modelling, pair distribution functions, line profile analysis, Williamson–Hall method, Rietveld method, diffuse scattering

## Abstract

A nanocrystalline Fe–1.8%Cr steel powder was tested as a reference material on more than five powder diffraction instruments and configurations, as well as with different data-analysis methodologies. The material, commercially available at low cost, was ground in a high-energy planetary mill to obtain homogeneous crystalline domain dimensions of 10 (2) nm, with size dispersion of 5 (1) nm and a nominal dislocation density of the order of 2.90 (2) × 10^16^ m^−2^. The powder is stable, easy to handle and suitable for preparing samples in any measurement geometry. It is well suited for testing the modelling of diffraction peak profiles, either individually or across the entire diffraction pattern, as in the Rietveld method. This paper reports the simple details for the production of the material and the analysis of the diffraction patterns collected with both laboratory and synchrotron beamline instruments, using X-rays of different energies. In particular, the screening of the data based on integral breadths (Williamson–Hall method), the analysis by whole powder pattern modelling and the analysis by the Rietveld method are shown. Aspects related to diffuse scattering and pair distribution function analysis are also discussed.

## Abstract 2

A nanocrystalline Fe–1.8%Cr steel powder is proposed as a reference material for testing different powder diffraction instruments and configurations, as well as different data-analysis methodologies.

## 1. Introduction

X-ray diffraction (XRD) has been known for many years as a technique for both structural and microstructural characterization of materials, with the latter more directly related to line profile analysis (LPA) (Scardi *et al.*, 2018 ▸; David *et al.*, 2010 ▸; McCusker *et al.*, 1999 ▸). Even though some LPA methods are described in textbooks (Dinnebier & Billinge, 2008 ▸; Dinnebier *et al.*, 2018 ▸), there is still active research, especially to integrate the study of profiles into the most used structural analyses, such as the Rietveld method (Scardi *et al.*, 2018 ▸). LPA is not a trivial or automated analysis, and requires theoretical and methodological skills if significant and reliable results are to be obtained. For this reason, it is useful to have reference materials that provide clear and well reproducible XRD spectra, that are stable and easily analysed with both commercial and more sophisticated research instrumentation, and that can be distributed to a potentially large number of users. This makes it possible to evaluate the quality and specificity of the instruments and methods of analysis used.

In this work, we propose a powder of an iron–chromium alloy (Fe–1.8%Cr) (Höganäs) (see Table S1 of the supporting information for chemical analysis), hereafter referred to as FeCr for simplicity, which, suitably plastically deformed by high-energy ball milling, has desirable characteristics for a reference material: the powder is monophasic, with a ferritic (b.c.c. – body-centred cubic) structure, which due to its high symmetry (space group *Im*3*m*) has intense and well spaced diffraction peaks. For this material, the strong plastic deformation is mainly linked to three effects: (*a*) the reduction of the coherent diffraction domains (crystallites) to dimensions of the order of 10 nm, with non-regular shapes that can however be approximated on average as equiaxial; (*b*) the formation of line defects, and the substantial absence of energetically unfavourable planar defects; and (*c*) the formation of grain-boundary structures, due to the fragmentation of the domains and the instability of the dislocations, which tend to slip across the domains to occupy the grain-boundary region, with only a fraction remaining trapped in the domains (Rebuffi *et al.*, 2016 ▸; Scardi *et al.*, 2017 ▸).

In this way it is possible to imagine – as has been verified with both electron microscopy and molecular dynamics (MD) simulations – that the line-broadening effects are substantially reduced to those due to the small dimensions of the domains, and to a fluctuation of the strain (‘microstrain’) caused by the remaining line defects and grain boundaries, as well as to grain–grain interactions (D’Incau *et al.*, 2007 ▸; Scardi *et al.*, 2017 ▸). As shown below, this allows a detailed analysis of the XRD pattern and provides information on the microstructure of the material in a simple way. The proposed material is also stable, due to the passivity effect attributable to the presence of chromium; the nanodomains are strongly aggregated, in particles of the order of tens of micrometres with limited oxidation, which in any case does not give measurable effects in the XRD powder pattern. The material is not subject to variations if pressed, having already been heavily work hardened, or incorporated with glues to produce tablets; it can be produced in large quantities, owing to the low cost of the base material and milling treatment, so as to allow a wide distribution.

In this work, we provide details on the preparation of the material and we show the results of the XRD pattern analysis that can be conducted with the whole powder pattern modelling (WPPM) approach (Scardi & Leoni, 2002 ▸), using the popular software *TOPAS* (Coelho, 2018 ▸; Scardi *et al.*, 2018 ▸). Comparison with the Williamson–Hall (WH) approach will also be shown, as it is often used for data screening or as a simplified, though rather approximate, tool for the microstructural analysis of powder patterns (Scardi *et al.*, 2004 ▸). The ball-milling methodology is based on fine-tuned experimental procedures applied for steel samples (D’Incau *et al.*, 2007 ▸). The results, and also the XRD patterns collected with different instruments, are available upon request to allow comparisons and checks for those who receive portions of this material, and also for those who just want to perform their own analyses of the FeCr data.^1^ This adds an educational value to the article.

## 2. Experimental details

The FeCr powder is an Astaloy CrA steel (containing 1.8 wt% Cr, see Table S1 for chemical analysis) (Hoganas, 2025 ▸). Batches of 17 g were ball milled in a Fritsch P4 planetary ball mill equipped with a stainless-steel vial (304 austenitic grade) of 80 ml capacity. A total of 25 balls (12 mm diameter) of 100Cr6 steel were used for the milling, for a ball-to-powder ratio of 10:1. Ethanol (Sigma–Aldrich) served as lubricant, corresponding to 1% of the powder’s weight. On the basis of previous work (D’Incau *et al.*, 2007 ▸), milling times of 8, 32 and 64 h were selected, stopping for 30 min every 4 h to avoid overheating. The final powder, proposed as a reference material, was ball milled for 64 h. The main disc rotation speed was set to 300 r min^−1^, with a planet-to-main-disc ratio of −1.8. A first batch of powder was prepared in order to allow the grinding tools to be run in, homogenizing the grinding media and reducing contamination. This batch was subsequently discarded and a second batch, free of visible contaminations, was used for the following analysis.

The XRD measurements employed several setups: (i) Bruker D8 Discover employing a Co *K*α target in Bragg–Brentano geometry and a 1D Lynxeye XE-T detector with 2° 2θ coverage; (ii) Rigaku PMG/VH equipped with a Cu target, graphite monochromator and zero-point detector, also in Bragg–Brentano geometry; (iii) synchrotron measurements obtained at ESRF, ID31 beamline [0.16563 (2) Å wavelength], using the Debye–Scherrer geometry and a Pilatus CdTe 2M 2D detector set at sample-to-detector distances (SDDs) of 1500 mm (in high-resolution mode) and 300 mm [for total scattering analysis (Terban & Billinge, 2022 ▸; Egami & Billinge, 2003 ▸)], with proper parallax and offset corrections performed (Marlton *et al.*, 2019 ▸); and (iv) ESRF again, ID22 beamline with 0.35434 (2) Å wavelength in its standard high-resolution mode, scanning the multi-analyser stage to 120° of 2θ to collect data suitable for pair distribution function (PDF) analysis (Fitch *et al.*, 2023 ▸). Laboratory measurements (i) and (ii) required the preparation of specimens for flat-sample geometry, for the reflection condition of the Bragg–Brentano geometry. For option (iii), the sample was sandwiched between Kapton foils of 1 mm thickness (see further details in the supporting information, Supplementary Note 1). For option (iv), the FeCr powder was loaded in a borosilicate glass capillary of 0.5 mm diameter. LaB_6_ (NIST SRM660c; Black *et al.*, 2020 ▸) served as the standard material for obtaining the instrumental resolution function in all setups, modelled using the fundamental parameter approach (FPA) (Cheary & Coelho, 1992 ▸) for laboratory data and using standard procedures defined for the synchrotron beamlines (Fitch *et al.*, 2023 ▸; Wang *et al.*, 2008 ▸). The Rietveld and WPPM analyses employed the *TOPAS 7* software (Coelho, 2018 ▸). PDF analysis was performed with both *TOPAS* and *PDFgetX3* (Juhás *et al.*, 2013 ▸; Billinge & Farrow, 2013 ▸; Peterson *et al.*, 2003 ▸). Blank measurements were also taken with the empty capillaries/supports for background correction. No absorption corrections were necessary for measurements (i) and (ii) in Bragg–Brentano geometry, while for the data collected in transmission, both (iii) and (iv), the absorption was low enough to be neglected.

Complementary scanning electron microscopy analyses were performed with a FEGSEM JSM-7001F (JEOL, Tokyo, Japan) instrument equipped with an energy-dispersive X-ray spectroscopy (EDXS) detector (INCA PentaFETx3, Oxford, UK). The energy of the electron beam was in the range 10–15 keV, with a working distance of 5–10 mm. The powder specimens were deposited on carbon tape. Samples for transmission electron microscopy (TEM) were prepared on ultra-thin carbon TEM carriers. The powder was therefore dispersed in ethanol. The samples were imaged by TEM on a Tecnai Osiris machine (Thermo-Fisher, Hillsboro, USA) operated at 200 keV under high-angle annular dark-field scanning TEM conditions. EDXS was applied to map the chemical composition. EDXS data were evaluated using the Thermo-Fisher *Velox 2* software package. Inductively coupled plasma atomic emission spectroscopy (ICP-OES) was performed in a Spectro Ciros instrument to verify the proportion of metals and impurities in the steel samples. Two samples were analysed: the pristine powder and the 64 h ball-milled powder. Details are given in Supplementary Note 2.

MD simulations were performed for a nanosphere of 12.5 nm diameter in a vacuum (simulation box size of 60 nm) using the *Large-Scale Atomic/Molecular Massively Parallel Simulator* (*LAMMPS*; Thompson *et al.*, 2022 ▸) with embedded-atom model potential (Mendelev *et al.*, 2003 ▸). The nanosphere was constructed using *VESTA* (Momma & Izumi, 2011 ▸, 2008 ▸) with a starting lattice parameter of 0.28731 nm. The Debye scattering equation was used to calculate the simulated total scattering patterns using the software *Debyer* (Wojdyr, 2020 ▸). These simulations served to corroborate the effectiveness of WPPM for the modelling of nanospheres and to obtain independent information on the local dynamic structure of b.c.c. Fe, necessary for the starting values of parameters in the modelling of the diffuse component. Details of these procedures can be found in the literature (Scardi & Malagutti, 2024 ▸; Malagutti *et al.*, 2025 ▸).

## 3. Line profile analysis methods

The conventional approach in XRD pattern fitting is based on empirical bell-shaped profile functions, with Lorentzian, Gaussian and Voigtian (convolution of Lorentzian and Gaussian) curves among the most popular (Coelho, 2018 ▸). The crystalline domain (or crystallite) size (CS) and microstrain (MS) can be empirically related to the peak width (full width at half-maximum, FWHM) or the integral breadth (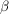) of each reflection in the line profile according to the Scherrer formula for CS and the tangent rule of Stokes & Wilson for MS (Cullity, 1978 ▸; Scardi *et al.*, 2004 ▸; Wilson, 1949 ▸). To separate CS and MS, the peak widths (FWHM or β) can be plotted as a function of the reciprocal-space variable 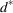 (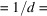

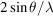
, with *d* as the interplanar distance, θ as the Bragg angle and λ as the radiation wavelength) according to the classical WH plot (Wilson, 1949 ▸; Scardi *et al.*, 2004 ▸):
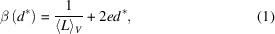
where β is in units of reciprocal space [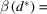

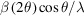
]. In a WH plot the intercept retrieves the inverse of 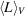, the volume-weighted mean column length (sometimes denoted as the effective CS or Scherrer size), while the slope gives the MS term 2*e*, double the maximum (upper limit) of a uniform strain distribution. If the strain follows a normal distribution, then 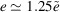, where 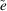 is the root-mean-square strain (RMSS) (Wilson, 1949 ▸).

The WH plot often shows deviations from linearity due to various factors, including the anisotropy of the elastic medium and strain field of the defects present. As shown by Stokes & Wilson (Wilson, 1949 ▸), this implies a dependence of 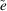 on *hkl*. Equation (1) can be modified to include anisotropy, by introducing the average contrast factor 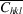, a polynomial function of the Miller indices dependent on the Laue group of the studied phase (Wilson, 1949 ▸). For cubic systems, like the b.c.c. ferritic steel in the present case, it can be shown that
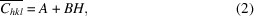
with 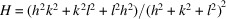 (Wilson, 1949 ▸). If 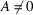, it is also possible to write
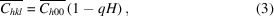
with 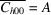 and 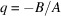. *A* and *B* (or 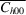 and *q*) can be treated as free (fitting) parameters, but if the elastic tensor of the material and the strain field of the defects are known, as for dislocations (see below), they can be calculated (Ungár & Borbély, 1996 ▸; Dragomir & Ungár, 2002 ▸; Ungár & Tichy, 1999 ▸; Scardi *et al.*, 2004 ▸). A modified WH (MWH1) expression can then be written as
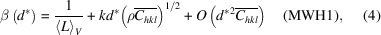
where *k* is a fitting parameter and ρ is the average dislocation density. As can be seen in equation (4), given the product of parameters it is not possible to obtain values of ρ without hypotheses on the values of the other parameters (*k* and the coefficients in 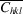), but it is possible to obtain the effective CS and follow the trend of the data for *q*, for example as the work hardening of the material increases. A further limitation of the method is the assumption of additivity of the CS and MS terms, which is empirical. In fact, it is possible to formulate alternative expressions such as, for example [see Scardi *et al.* (2004 ▸) for details],
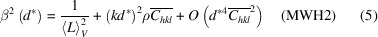
and
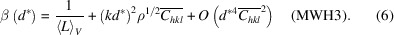
Which expression to use usually depends on which one best fits the case-study data. Literature results for similar systems indicate MWH3 as the most suitable (Scardi *et al.*, 2004 ▸; Wilson, 1949 ▸), an aspect that is addressed later in the context of this work. However, the WH method in its various forms [equations (1), (4), (5) and (6), and others (Scardi *et al.*, 2004 ▸)] is burdened by several (over)simplifying assumptions, in addition to the simple additivity of the CS and MS terms. This suggests using the method mainly for a preliminary screening of the data, with results to be carefully considered and possibly confirmed by other more reliable and complete methods like the WPPM approach discussed below. For example, if the microstructural features of the material are direction independent (*hkl* independent), we expect that equation (1) will follow the experimental data well. In the case of interest, a ferritic (b.c.c.) steel, we expect instead a strong anisotropy of the MS term, which can be recognized as shown in the following, using the MWH in one of the possible forms [equations (4), (5) and (6)].

To treat CS and MS effects reliably it is advisable to model the entire diffraction profile for all reflections present in the experimental pattern. This is what the WPPM approach does, through various possible models of crystallite shape and size dispersion, as well as different types of microstructural defects. Details are described in the literature (Scardi & Leoni, 2001 ▸, 2002 ▸; Leoni *et al.*, 2006 ▸; Scardi, 2020 ▸), including the possibility of using this approach in *TOPAS*, one of the most popular Rietveld analysis software packages (Scardi *et al.*, 2018 ▸; Coelho, 2018 ▸).

WPPM relies on equations for crystallite shape and size distribution retrieved after Fourier transformation (FT) of the line profile contributions (Jagodzinski, 1963 ▸). Since microstructural and other instrumental aberrations act as a convolution on the broadening of the line profile, in FT these contributions appear multiplied together. The instrumental profile can be modelled separately, with parameters obtained using a standard reference material. In *TOPAS* this is done according to the cited FPA (Cheary & Coelho, 1992 ▸), considering the known geometry of the experimental setup, with the slits and other optical components used. Synchrotron aberrations are generally smaller and are adjusted using Thompson–Cox–Hastings-based functions (Thompson *et al.*, 1987 ▸).

For the microstructural features of the case in question, also on the basis of experimental evidence of similar systems (D’Incau *et al.*, 2007 ▸), it is appropriate to assume an equiaxial average shape for the crystallites, and hence a spherical model, with diameters (*D*) dispersed according to a lognormal distribution
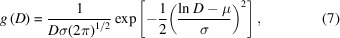
with lognormal mean (μ) and variance (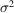). The distribution moments are defined as
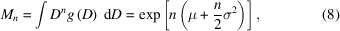
so that the arithmetic mean is
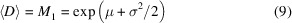
and the standard deviation is
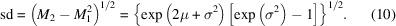
According to this model, the FT of the line profile due to the CS component is given by
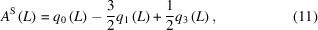
where *L* is the Fourier length (or distance) within the crystallite and
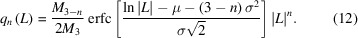
To account for the MS effect of dislocations on the line profile, the Krivoglaz–Wilkens model is most commonly employed (Krivoglaz & Ryaboshapka, 1963 ▸; Krivoglaz *et al.*, 1983 ▸; Wilkens, 1970 ▸; Martinez-Garcia *et al.*, 2009 ▸). This gives the MS for a distance *L* along [*hkl*] (Warren, 1990 ▸) in the form
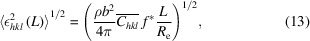
where 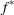 is the function defined by Wilkens (1969*a* ▸,*b* ▸), *b* is the modulus of the Burgers vector of the dislocation system and 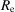 is the effective outer cut-off radius of the dislocation strain field. Average contrast factor coefficients can be calculated for different types of dislocations. Considering edge and screw dislocations in cubic systems (Wilkens, 1969*a* ▸,*b* ▸; Scardi *et al.*, 2018 ▸; Martinez-Garcia *et al.*, 2009 ▸), and a mixing parameter 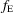 (usually constrained as 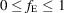), the coefficients of 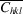 in equation (2) can be written as
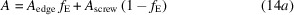
and
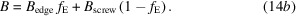
Values of 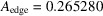, 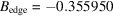, 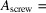 0.307288 and 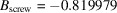 are used for b.c.c. Fe (Dinnebier & Billinge, 2008 ▸). The FT of the line profile due to the MS component is given by
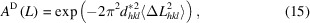
where 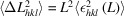 is the mean-square displacement. Therefore, in the analysis of the diffraction pattern of FeCr ball-milled powders, the free parameters to be refined together with the characteristic ones of the Rietveld method are μ and σ of the lognormal distribution and ρ, *R*_e_ and *f*_E_ for the MS. The WPPM analysis thus provides detailed information on the diffraction domains, their dispersion and characteristics of the defects, here assumed to be dislocations.

However, it is possible that other defects contribute to the MS line broadening, such as grain boundaries, nanocrystal surfaces, cell-parameter fluctuations, grain–grain interactions *etc*. In this case, equations (13) and (15) can still be used to describe the line-broadening effect, but ρ should only be considered as an upper limit for the density of line defects, *i.e.* refined ρ values tend to overestimate the actual dislocation density. The LPA results can be more conveniently displayed in a Warren plot (Warren & Averbach, 1950 ▸), which reports the root-mean-square displacement, 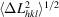, as a function of *L* for various crystallographic directions, in order to highlight the anisotropy effect (Warren & Averbach, 1950 ▸; Scardi *et al.*, 2018 ▸). This type of representation, as shown in detail later, may be preferable because it has more general validity, independent of the specific defects and contributions to the MS line broadening.

## 4. Results and discussion

Below we show FeCr powder patterns collected with different instruments, both commercial laboratory ones and those available at synchrotron radiation facilities. This allows us to show the characteristics of the powder and how it can be flexibly used with instruments based on different configurations.

### 4.1. Ball-milling evolution

**Figure 1:**
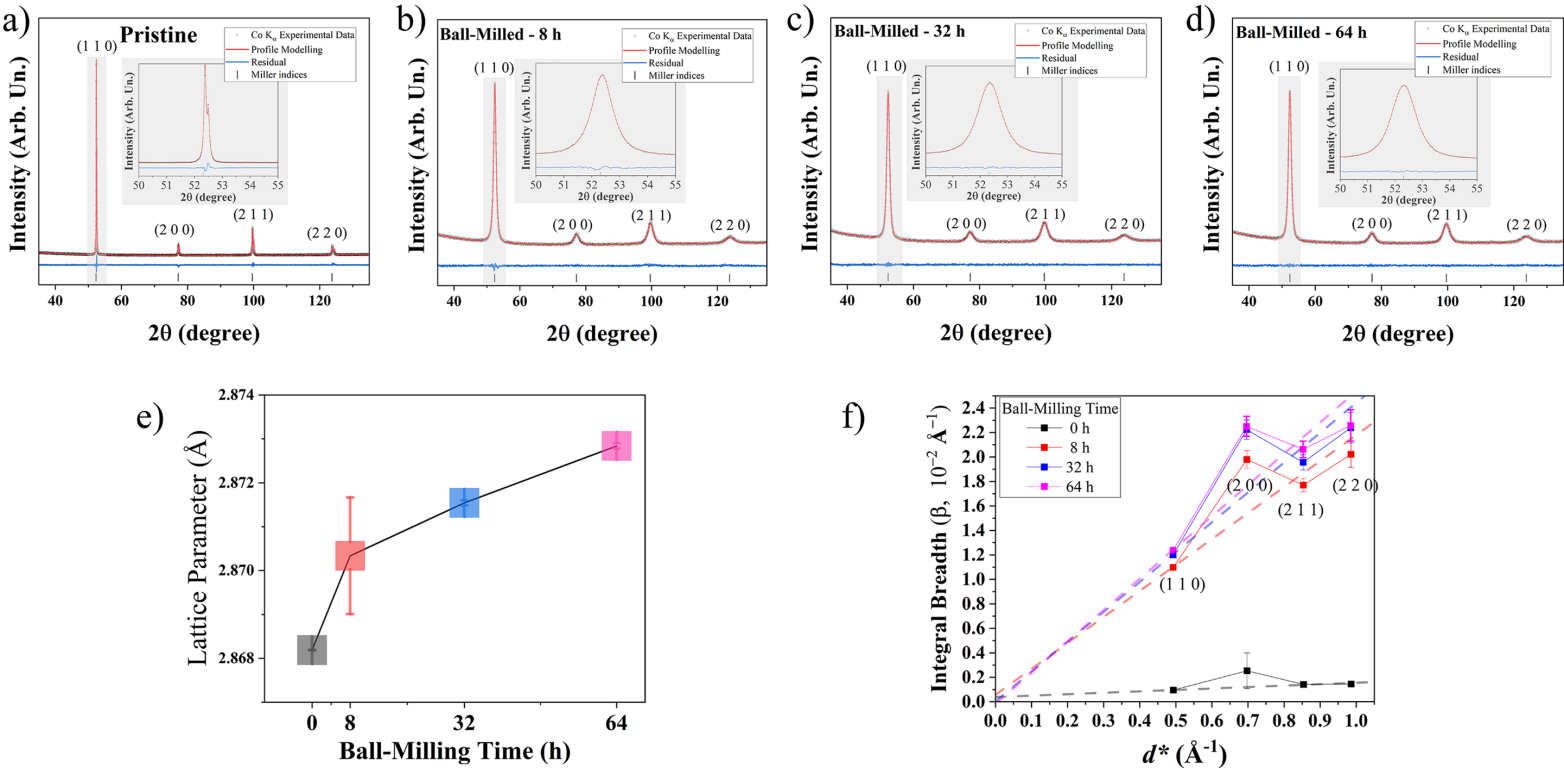
XRD patterns obtained with Co *K*α radiation using setup (i). Data modelled according to the Pawley method, with Voigtian line profiles: (*a*) pristine FeCr powder, and after (*b*) 8 h, (*c*) 32 h and (*d*) 64 h ball milling. The insets show an amplified view of the 110 reflection. Effect of the ball-milling time on (*e*) lattice parameter and (*f*) integral breadth in the traditional WH plot [dashed lines refer to equation (1), see main text for details].

FeCr powder patterns for increasing ball-milling time are shown in Fig. 1 ▸. The data were collected using a laboratory diffractometer with Co *K*α radiation, setup (i), involving the two characteristic *K*α_1_ and *K*α_2_ emission profiles, a feature visible for the pristine powder in Fig. 1 ▸(*a*). Co *K*α radiation wavelength, λ = 1.788969 Å (Hölzer *et al.*, 1997 ▸), is relatively long among those commonly used for XRD. As such, it provides well separated diffraction peaks, but with a limited range of 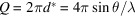, and therefore few diffraction peaks, only four in the present case. This condition limits the information that can be extracted from experimental data. The pattern was therefore modelled with the Pawley method (Pawley, 1981 ▸): the peak positions are constrained by the lattice parameter, freely refined together with the peak intensities and the parameters of the Voigt functions that model the line profiles, with a fifth-degree Chebyshev polynomial for the background. Flat and featureless residuals [bottom line, difference between observed (circle) and model (line) data], especially for the ball-milled sample patterns of Fig. 1 ▸, demonstrate the good quality of the modelling.

A measurable effect of ball milling is the progressive increase of the lattice parameter with the grinding time shown in Fig. 1 ▸(*e*). The literature on similar cases indicates various mechanisms underlying the observed trend, like the increase in oxygen and vacancy solubility with decreasing grain size [as per the Gibbs–Thomson equation (Liu *et al.*, 1994 ▸)] and the high density of dislocations at grain boundaries (Naza­rov *et al.*, 1996 ▸). Further information on the evolution of the ball-milling treatment is provided by the integral breadths in the WH plot of Fig. 1 ▸(*f*). The systematic deviation from the linear trend due to the anisotropy of the elastic medium and defects can be treated with the MWH1–MWH3 models [equations (4), (5) and (6)], an aspect that will be shown later. For now, a visual screening of the data in Fig. 1 ▸(*f*) already provides us with useful information: as milling time increases the trend of the integral breadth shifts upwards and increases in slope, characteristics that according to equation (1) are related to the inverse of the effective CS and the MS, respectively. Ferritic steels are ductile materials, which deform plastically in the early stages of ball milling, strongly reducing the CS until reaching a saturation value. Prolonged times lead to an increase in defect density, but annealing effects can also be produced, which partially cancel or at least counteract the work hardening (Liu *et al.*, 1994 ▸). It is therefore evident that grinding decreases the CS while it increases defect density and associated MS, but the effect tends to saturate for long milling times. Since we are interested in discussing a reference material for LPA, with evident CS and MS effects, the following more detailed analysis of the line profiles focuses on the powder milled for 64 h.

**Figure 2:**
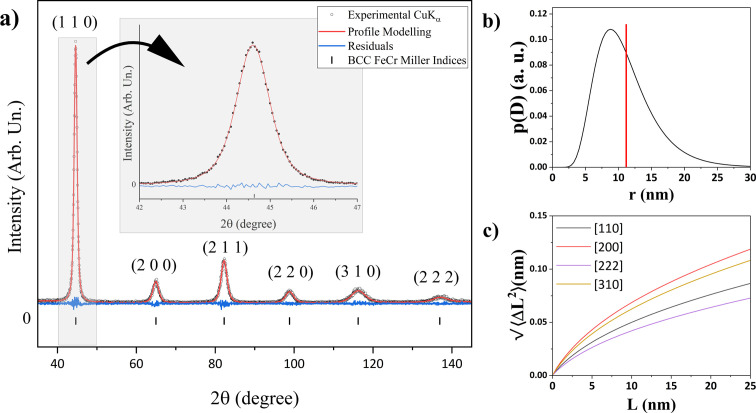
(*a*) XRD pattern measured with Cu *K*α radiation: experimental data (circles), profile modelling (red line) and residual (blue line below). (*b*) Diameter distribution of spherical FeCr domains, with mean value 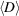, red bar, and (*c*) Warren plot for the directions [110], [200], [310] and [222]. See main text for details.

The XRD data and modelling for the 64 h FeCr sample using setup (ii) are presented in Fig. 2 ▸(*a*). See Fig. S2 of Supplementary Note 3 of the supporting information for the LaB_6_ standard (NIST SRM 660c) powder pattern with the modelling of the instrumental contribution according to the FPA (Cheary & Coelho, 1992 ▸). The Cu *K*α radiation used in setup (ii) has λ = 1.540570 Å (~8048 keV, *K*α_1_ component) (Deutsch *et al.*, 1995 ▸), giving 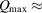 8 Å^−1^ – the maximum value of *Q*, such that reflections up to 222 can be observed. The pattern was analysed according to the Rietveld method, using *TOPAS* supported by WPPM-based macros for line profiles [Section 3 and Scardi *et al.* (2018 ▸)]. The structure used is the conventional one of b.c.c. Fe (ICDD PDF4 database record number 00-006-0696) (Gates-Rector & Blanton, 2019 ▸). The residual in Fig. 2 ▸(*a*), relatively flat and featureless, confirms also in this case the good quality of the modelling, the results of which are reported in Table 1 ▸. The average domain size is around 11 nm, with a dispersion shown by the distribution of Fig. 2 ▸(*b*), with a standard deviation of 4.5 nm. The presence of domains smaller than ~5 nm or larger than ~20 nm is very unlikely.

If the MS was entirely attributed to dislocations, the density would be 3 × 10^16^ m^−2^: a high value due to the strong work hardening caused by the long ball milling. The value of the effective outer cut-off radius was set to the average domain size to limit the free parameters and retrieve better starting values for 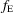 (Leonardi & Scardi, 2015 ▸, 2016 ▸), but left to refine in the following interactions. The character of the dislocations [the mixing parameter 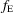 in equation (14)] is towards the edge type (62%). This is consistent with the effects of prolonged ball milling, which promotes thermally activated recovery processes. These include dislocation annihilation, which occurs especially for screw-type cross slips, resulting in a prevalence of edge-type dislocations. The fact that the value of 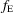 is well within the limits between 0 and 1 (Section 3) demonstrates that the anisotropy model is applicable to the case study.

As discussed in Section 3, the MS effect is best represented by Warren plots, shown in Fig. 2 ▸(*c*) for different crystallographic orientations. The largest displacement is along [*h*00] and the smallest along [*hhh*], with all other directions in between. This trend follows the elastic anisotropy of iron, which, like many cubic metals, has a stiff direction along [*hhh*] and a soft direction along [*h*00] (D’Incau *et al.*, 2007 ▸). Further details are discussed later, with results obtained with synchrotron radiation.

### 4.2. Synchrotron data analysis

The use of synchrotron radiation has several advantages over laboratory sources, most notably an intense flux of high-energy X-rays to reach higher 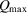. This allows for more reliable refinements of the structural parameters, including the thermal effects, described by the Debye–Waller (DW) factor and the corresponding DW coefficients (Scardi & Malagutti, 2024 ▸; Malagutti *et al.*, 2025 ▸). Such effects are not well observable with setups (i) and (ii) due to the strong absorption and short *Q* range, which includes few Bragg peaks. Nevertheless, as shown below, the LPA results are surprisingly stable and comparable to those obtained from laboratory-scale instruments.

**Figure 3:**
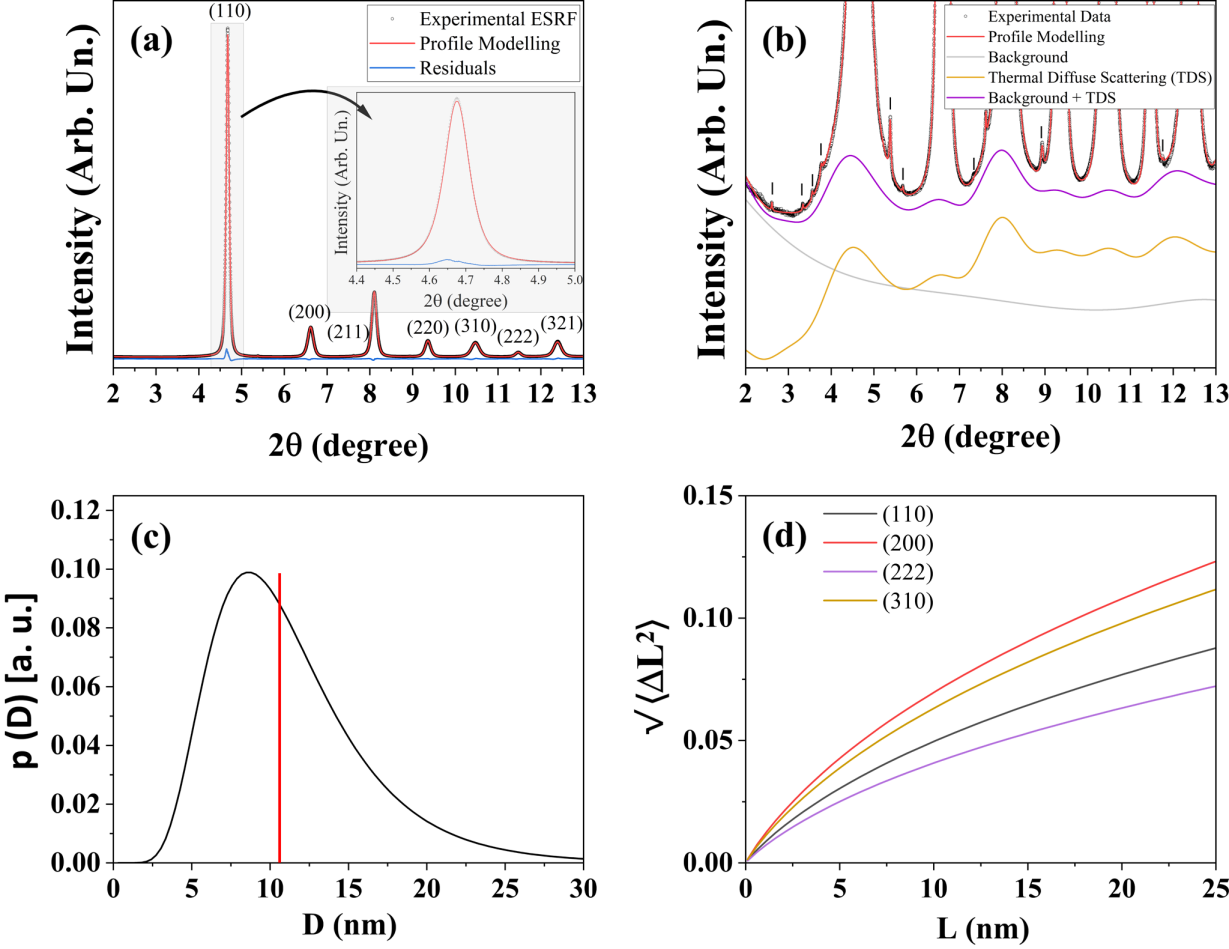
(*a*) XRD pattern for the Fe_1.8_Cr steel milled for 64 h obtained at the ID31 beamline at the ESRF synchrotron for 1500 mm SDD. Black dots correspond to the experimental data, red lines to the profile modelling and blue lines to the residuals. (*b*) Zoomed part of the diffuse component of the previous item (*a*). The grey line corresponds to the background, the yellow line to the TDS and the purple line to the sum of all terms above. (*c*) Distribution of the crystalline domain diameters. (*d*) Warren plots. Each colour represents reflections and corresponding crystallographic directions, with indices indicated in the inset.

Using synchrotron radiation in setup (iii), the X-ray energy increases to 75 keV, which allows exploration of the reciprocal space up to 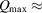 10 Å^−1^ for the high-resolution mode (SDD of 1500 mm). Hence, reflections up to 321 are visible and modelled, as shown in Fig. 3 ▸(*a*) for the 64 h ball-milled FeCr powder. As reported in Table 1 ▸, the WPPM analysis gave *R*_wp_ ≃ 1.5% and goodness of fit (GoF) ≃ 6.2, with relatively low residual features [blue line in Fig. 3 ▸(*a*)].

Among the benefits of synchrotron radiation XRD, the high photon energy can be exploited to minimize or eliminate absorption effects, which are difficult to correct. In fact, absorption is made negligible by using capillaries with fillings that achieve beam transmission of 95% or more. These conditions allow for reliable modelling of the diffuse part, *i.e.* of any contribution that falls out of the perfect periodicity of the crystallite domain. The diffuse component contains information on the local dynamic properties of materials. Therefore, it is called thermal diffuse scattering (TDS), even if it also includes static disorder effects. Its modelling using the Sakuma approach (Sakuma, 1995 ▸; Scardi & Malagutti, 2024 ▸; Wada *et al.*, 2012 ▸; Malagutti *et al.*, 2025 ▸) is shown in Fig. 3 ▸(*b*), yellow line.

The use of the TDS model in the Rietveld refinement allows for a more precise estimate of the DW coefficient since both diffuse and Bragg contributions are considered simultaneously, resulting in a DW coefficient of 0.352 (2) Å^2^. The TDS model used considers that the atomic thermal displacements, especially for the first neighbours, are not random and independent. For this reason, correlation coefficients of atomic displacements (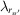) are introduced for each pair of atoms *s* and 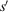 at distance *r*. This is responsible for the TDS oscillatory behaviour beneath the Bragg peaks [see Fig. 3 ▸(*b*)]. Reliable starting points for the 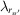 were obtained using MD simulation by fitting of the radial PDF in a procedure similar to that reported by Scardi & Malagutti (2024 ▸). Values are displayed in Fig. S3 of Supplementary Note 4.

**Figure 4:**
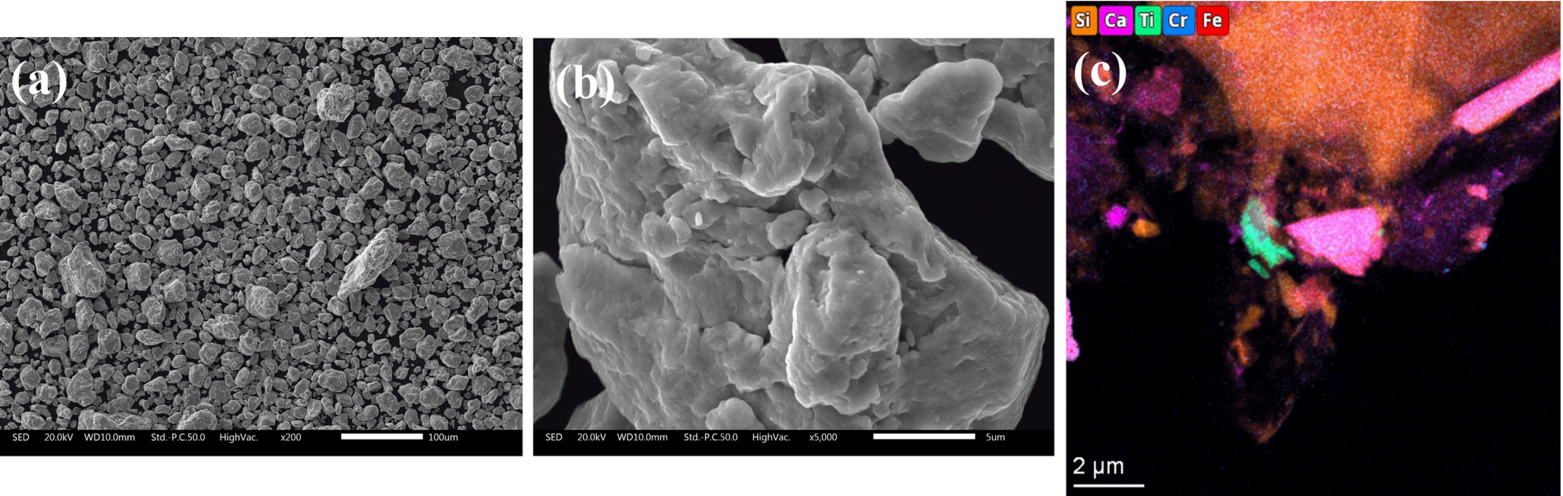
Morphology of the FeCr 64 h sample obtained via scanning electron microscopy with different magnifications, from (*a*) 200× to (*b*) 5000×; nanocrystalline domains appear strongly agglomerated in much larger micrometre-scale particles. (*c*) EDXS analysis. See main text for details.

The high signal-to-noise ratio brings out the signal of a small fraction of impurities [see details in Fig. 3 ▸(*b*)]. The signal of these impurities is barely visible and corresponds to less than 0.2% contribution to the total peak area analysed. The scanning electron microscopy and EDXS analysis presented in Fig. 4 ▸ were employed for identification. The EDXS analysis revealed that Al, Cu, Si and Ni are present in the sample, but correspond to less than 1% in atomic fraction and do not increase with milling time (see Table S1 of Supplementary Note 5). Further TEM-EDXS analysis shows the presence of Ca and Ti in isolated spots in the sample, and ICP-OES analysis revealed trace amounts of Mn (see Table S2 of Supplementary Note 5). No match was obtained by employing the PDF4+ database (Gates-Rector & Blanton, 2019 ▸) for phase identification. However, the contribution of impurities is deemed to be insignificant and does not correlate with the LPA of the FeCr powder.

The CS distribution and Warren plots obtained using this configuration are given in Figs. 3 ▸(*c*) and 3 ▸(*d*), respectively. As also shown by the results in Table 1 ▸, the microstructural parameters are surprisingly similar to those obtained with Cu *K*α radiation and a conventional laboratory instrument [setup (ii)]. Despite the few Bragg peaks observed in the setup (i) pattern, a WPPM-based analysis (results shown in Table 1 ▸) also leads to very similar results. The FeCr powder therefore seems suitable for comparing LPA results from different measuring instruments and geometries.

**Figure 5:**
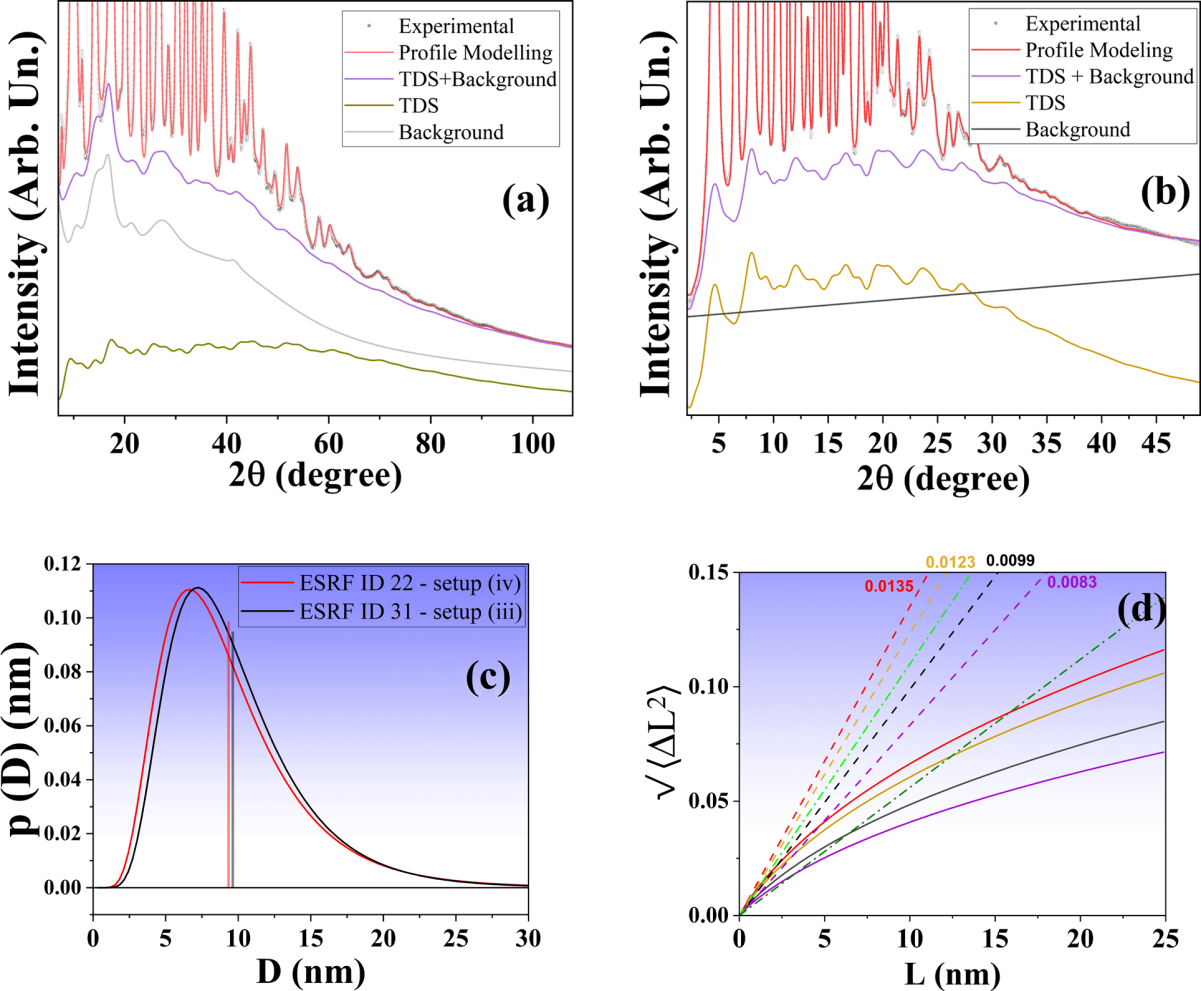
XRD modelling of the FeCr powder ball milled for 64 h measured at the (*a*) ESRF ID22 beamline [setup (iv)] and (*b*) ESRF ID31 beamline [setup (iii)]. The dots represent the experimental data, the red line represents the profile modelling, the green (*a*) and yellow (*b*) lines correspond to the TDS contribution, grey is the background, and purple is the sum of the TDS and background. (*c*) CS distribution obtained by WPPM for both setups. (*d*) Warren plots obtained via the Wilkens model of equation (13) and setup (iv) data. Dashed lines show the initial tangents for the different directions and the corresponding true strain values {ε = 0.0135 [200], 0.0123 [310], 0.0099 [110] and 0.0083 [222]}. The dash–dot lines represent the effective Warren plots obtained via PDF analysis (Section 4.3). Light green corresponds to setup (iii) and dark green to setup (iv).

To further validate the FeCr powder as a reference material for LPA, additional measurements were performed with an increased range of 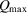 of 30 and 28.6 Å^−1^ for setup (iii) (with 300 SDD) and setup (iv), respectively. Such high-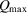 measurements are ideal to study the TDS since the entirety of the diffuse component is recorded. The powder pattern modelling is shown in Figs. 5 ▸(*a*) and 5 ▸(*b*), for setups (iv) and (iii), respectively, with modelled parameters reported in Table 1 ▸. Residuals are given in Figs. S4 and S5 of Supplementary Note 4. In reciprocal space, the TDS is modelled again following the Sakuma approach (Malagutti *et al.*, 2025 ▸; Scardi & Malagutti, 2024 ▸; Sakuma, 1995 ▸), as shown by the yellow lines of Figs. 5 ▸(*a*) and 5 ▸(*b*). As stated before, the advantage of this approach is the retrieval of the DW coefficient with more precision, since both diffuse and Bragg scattering are modelled simultaneously. The refined DW values, 0.336 (4) Å^2^ with setup (iii) and 0.328 (3) Å^2^ with setup (iv), are in good agreement with the result obtained with setup (iii) with an SDD of 1500 mm. However, this result is more reliable for the *Q*-space extension of the data, which allows, as for the subsequent PDF analysis, one to reliably isolate the background contribution. On this basis, it is suggested to keep this contribution fixed to a value of around 0.33 Å^−2^ when using this material as a standard. The average CS value is close to that of the previous synchrotron measurement, with a lognormal distribution plotted in Fig. 5 ▸(*c*) for both setups. The same goes for the Warren plots of Fig. 5 ▸(*d*), with trends superimposable to those of setup (iii) in Fig. 3 ▸.

In addition to the anisotropy already commented on, further information is provided by the slope of the lines drawn from *L* = 0 to any value *L*, which corresponds to the RMSS averaged over columns of unit cells of length *L* along the direction considered (Warren & Averbach, 1950 ▸). The initial slopes of the curves drawn in Fig. 5 ▸(*d*) as dashed lines are of particular interest, providing the true strain. Considering the strong work hardening undergone by the powder, this strain can be taken as the yield strain (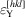), from which it is possible to derive an estimate of the yield stress (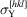) as
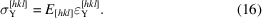

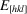
 is Young’s modulus in the 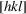 direction, which for cubic structures is given by
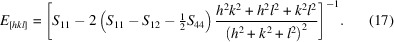

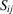
 are the coefficients of the elastic compliance tensor, the inverse of the stiffness tensor 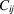, whose coefficients are 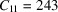
 GPa, 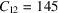
 GPa and 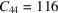
 GPa for iron (Rayne & Chandrasekhar, 1961 ▸). The resulting stress (see Table 2 ▸) is about 2 GPa. This is in agreement with literature values for 10 nm steel nanopowders (Benson *et al.*, 2001 ▸; Yu *et al.*, 2011 ▸), obtained assuming the 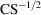 (Hall–Petch) relationship between yield stress and average crystallite size, CS (Hall, 1951 ▸; Petch, 1953 ▸). In addition, the resilience can be estimated via (Callister & Rethwisch, 2018 ▸; Ho *et al.*, 1972 ▸)
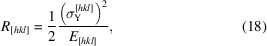
with values given in Table 2 ▸. The average resilience is ~11 MJ m^−3^, significantly higher than that of pure b.c.c. Fe [~0.12 MJ m^−3^ (Callister & Rethwisch, 2018 ▸; Ho *et al.*, 1972 ▸)], medium-carbon steels [~0.3–0.9 MJ m^−3^ (Ashby & Jones, 2012 ▸)] and spring steel [~4.9–7.2 MJ m^−3^ (Hertzberg, 1996 ▸)] due to the nanometric characteristics of the FeCr steel powders. Performing mechanical stress/strain tests on isolated nanocrystals is remarkably challenging, so the methodology illustrated here can be used to evaluate mechanical properties that would otherwise be rather difficult to measure.

### 4.3. PDF analysis

The ESRF ID22 and ID31 data are also suitable for deriving the PDF, taking advantage of the data range with high 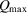 (Takeshi & Billinge, 2012*b* ▸; Terban & Billinge, 2022 ▸). PDFs are mainly used to study the local structural properties of materials, and some aspects of their microstructure. Microstructural effects influence two aspects of the PDF peaks, with a reduction in amplitude due to the CS effect and a broadening caused by the MS (Takeshi & Billinge, 2012*a* ▸; Beyer *et al.*, 2022 ▸; Olds *et al.*, 2015 ▸). The CS contribution is represented by the shape function [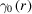], which is ~1 for short pair distances (*r*) and decreases to 0 for large values of *r*. In the PDF modelling, 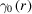 multiplies the reduced PDF, defined as
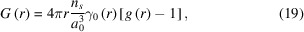
where 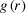 is the atomic PDF, 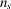 is the number of atoms per unit cell and 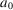 is the average lattice parameter. The shape factor is obtained by an orientational average of the common volume function (Farrow & Billinge, 2009 ▸; Guinier & Fournet, 1955 ▸), which for a distribution of spheres is equivalent to equation (11), written for *r* instead of *L* (Gamez-Mendoza *et al.*, 2017 ▸):
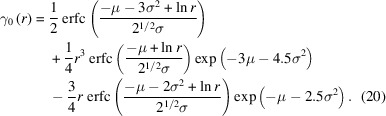
For the peak profile modelling, Gaussian functions are the most commonly used in PDF software such as *PDFGui* and *TOPAS* (Farrow *et al.*, 2007 ▸; Coelho, 2018 ▸). This function is defined as (Thorpe *et al.*, 2002 ▸; Beyer *et al.*, 2022 ▸)
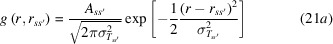
and
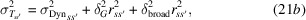
where 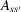 is a scaling factor for the pair of atoms *s* and 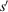 with pair distance 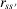, 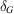 is the MS contribution, 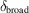 is the broadening due to the finite *q* step in reciprocal space, and 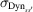 is the dynamic term, equivalent to the mean square relative displacement (MSRD).

Currently, PDF analysis does not include models such as the Krivolgaz–Wilkens of equation (13), which takes into account the direction-dependent behaviour of the MS. However, the sum of the standard deviation proposed by Lucks *et al.* (2001 ▸) proved to be more effective than equation (21*b*[Disp-formula fd23]) in the present case study. This implies writing the total variance as^2^
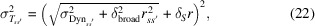
where 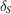 is equivalent to 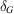, which corresponds effectively to the RMSS of a Gaussian strain distribution.

**Figure 6:**
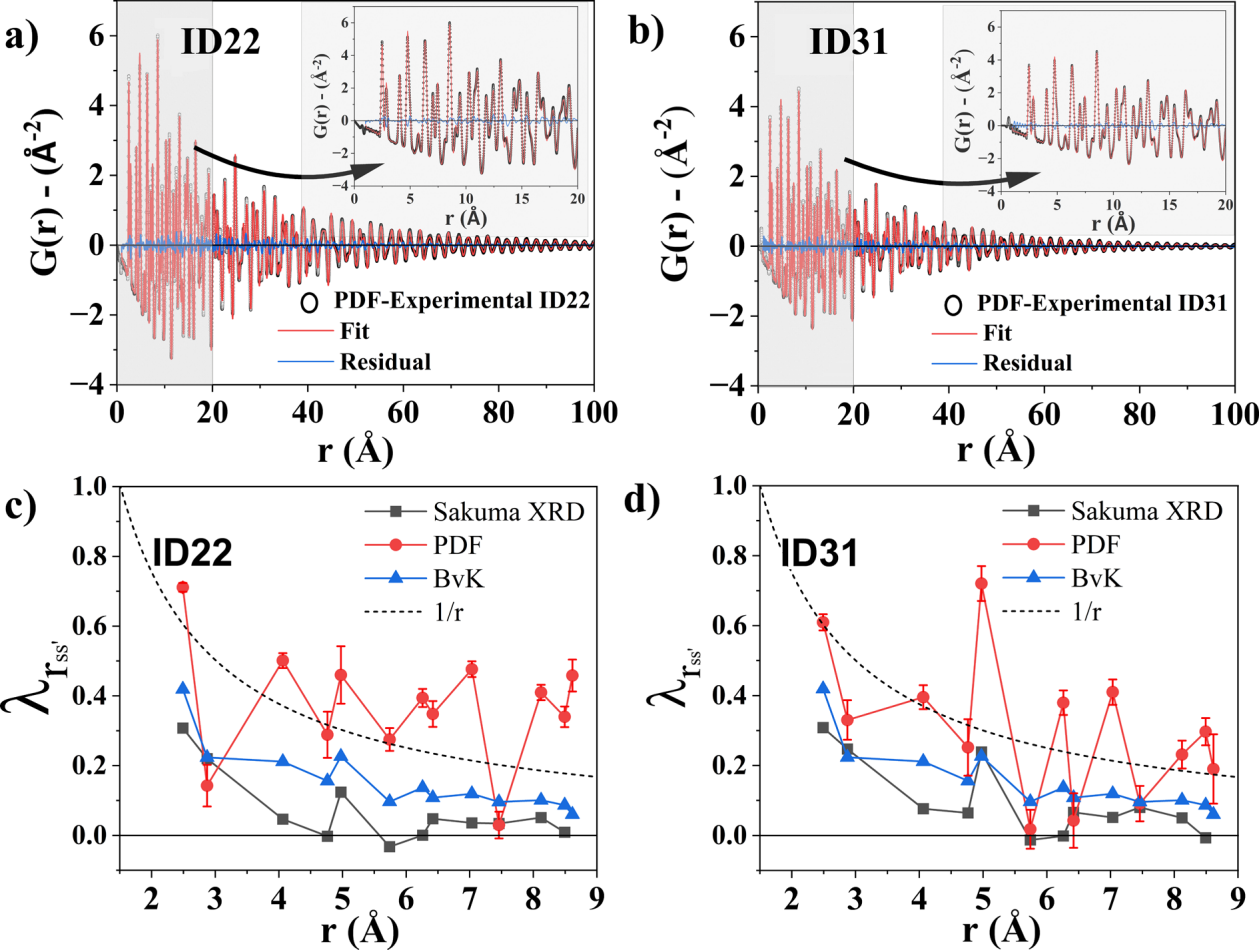
PDF fitting of the data collected at the (*a*) ID22 ESRF beamline [setup (iv)] and (*b*) ID31 ESRF beamline [setup (iii) – SDD of 300 mm] for the Fe_1.8_Cr sample ball milled for 64 h. (*c*), (*d*) Correlation coefficients estimated via PDF analysis (red circles), the Sakuma TDS model (black squares) and BvK values obtained from Jeong *et al.* (2003 ▸) (blue triangles) are shown for (*c*) setup (iv) and (*d*) setup (iii) – SDD of 300 mm. The dashed lines represent the empirical 1/*r* relation for the correlation coefficients. The fitted models in (*a*) and (*b*) show the model with a 1/*r* dependence relation.

The PDF modelling from the ESRF ID22 and ID31 datasets [setups (iv) and (iii) with an SDD of 300 mm] are shown in Figs. 6 ▸(*a*) and 6 ▸(*b*), respectively. The refined parameters are reported in Table 3 ▸. Details on the instrumental broadening and damping corrections are given in Supplementary Note 6, where the LaB_6_ NIST sample (Black *et al.*, 2020 ▸) was employed as standard. Regarding MS, the refined 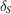 value from the PDF analysis is 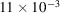 and 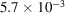 for Figs. 6 ▸(*a*) and 6 ▸(*b*), respectively. These values correspond to linear trends with slope 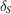 in the Warren plot, as shown by the dash–dot lines in Fig. 5 ▸(*d*). Although these data cannot describe the anisotropy effect and the nonlinear behaviour, the MS is consistent in order of magnitude with the values obtained in reciprocal space with the Rietveld method for the ID31 measurements (Section 4.2).

As already stated, thermal motion is not random, especially for the closest atoms. In this regard, it is possible to adopt a model for the dynamic term that includes the correlation of atomic displacements of pairs of atoms (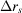 and 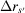) (Scardi & Malagutti, 2024 ▸; Malagutti *et al.*, 2025 ▸):
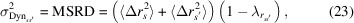
where
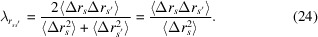
The second equation for the correlation coefficients (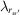) is valid for the specific case study of a single-element phase. These values are proportional to the number of neighbours in the coordination shell, the strength of the bond between the atoms and the distance between them (Wada *et al.*, 2012 ▸; Makhsun *et al.*, 2013 ▸). Therefore, different crystalline structures will present characteristic trends and magnitude with pair distance *r*. The magnitude of the correlation coefficients is intrinsically linked to the force constants, especially for the nearest neighbours (Makhsun *et al.*, 2013 ▸; Wada *et al.*, 2012 ▸), which means that the larger the 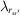, the greater the inter­atomic force between the pairs.

The 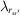 coefficients obtained from the PDF analysis are plotted in Figs. 6 ▸(*c*) and 6 ▸(*d*) for setups (iv) and (iii) with an SDD of 300 mm, respectively. Comparing results with the canonical work of Jeong *et al.* (2003 ▸) in this field, the trends of 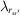 obtained from PDF analysis do not present any similarity to that of the Born–von Karman (BvK) model (blue triangle symbols in Fig. 6 ▸), and are significantly shifted to higher values. In the same figure, black square symbols correspond to the 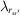 values obtained with the Sakuma TDS model used in the reciprocal-space data analysis (Fig. 5 ▸). These are in better agreement with the BvK model, both in trend and magnitude. The BvK model is still an approximation considering harmonic vibrational modes, and the approach of independently refining the coefficients 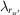 provides a more flexible way to account for the correlated motion. However, the fact that 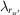 closely follow a physical model such as BvK confirms that it is possible to study correlation effects with a reciprocal-space approach employing the Sakuma strategy.

Other approximate methods, such as the one based on the Debye lattice model (Debye, 1912 ▸), are also used to model the 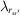 values in PDF analysis (Chen *et al.*, 2023 ▸). They can exhibit a 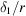 or 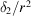 dependence on the pair distance, with the former typically used for data collected at temperatures above the Debye temperature and the latter for temperatures below it. The PDF fitting reported in Figs. 6 ▸(*a*) and 6 ▸(*b*) made use of the 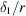 dependence, with 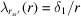 plots given by the dashed lines of Figs. 6 ▸(*c*) and 6 ▸(*d*). The 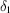 values are reported in Table 3 ▸.

The discrepancy between the 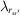 values obtained via PDF and those via analysis of the powder pattern in reciprocal space arises from the intrinsic characteristics of the two approaches, as well as from some aspects of the data modelling. In the PDF analysis, the MS and the dynamic components of the displacement are both extracted from the PDF peak widths. Since no model is currently available to account for the effect of dislocation strain, we can only add the linear-dependence term of equations (21*b*[Disp-formula fd23]) or (22) to the standard deviation of the PDF peaks, assuming an isotropic MS. As one can observe in the Warren plots of Fig. 5 ▸(*d*) and Section 4.2, b.c.c. steel presents a high degree of RMSS anisotropy, which is especially accentuated by the ball milling. This results in direction-dependent peak widths, different for each atomic pair in the PDF. Since the dynamic contribution, in the form of the correlation coefficients 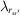, is also direction dependent [see Figs. 6 ▸(*c*) and 6 ▸(*d*)], the lack of an anisotropic MS model means that most of the anisotropy in the PDF peak broadening is accounted for by the dynamic factor. This leads to overestimation of the correlation coefficients derived from the PDF analysis, as shown in Fig. 6 ▸. Regarding the other parameters of interest, the DW coefficient (between 0.27 and 0.37 Å^2^, see Table 3 ▸) is close to the values obtained by reciprocal-space data analysis (see Table 1 ▸), whereas PDF analysis with equation (20) gives a CS from 7 to 9 nm, with a 6 to 8 nm standard deviation, significantly different from WPPM analysis. Therefore, further advancements in modelling microstructural effects are essential for the PDF analysis, and the FeCr powder can be used as a reference for such developments, especially by modelling the dislocation effect. In addition, further studies should focus on simultaneously modelling PDF and reciprocal-space data, for a comprehensive structural and microstructural analysis. This approach would leverage the visualization strengths of PDFs to elucidate local dynamics and incorporate WPPM for detailed microstructural characterization, holding a potential to bridge gaps between local structural and microstructural properties.

### 4.4. Williamson–Hall plot

The integral breadth (β) of the diffraction peaks is independent of the LPA approach employed, and after the deconvolution of the instrumental contribution, made possible by *TOPAS*, and the appropriate conversion into reciprocal-space (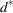) units, β is also independent of the instrumental setup. Integral breadths can therefore be used not only for data screening, as in Fig. 1 ▸(*f*), but also for a rapid comparison between different datasets, instruments and LPA strategies. All β values from measurements performed with different configurations [(i)–(iv) in Section 2] are reported in Table S4 of Supplementary Note 7.

**Figure 7:**
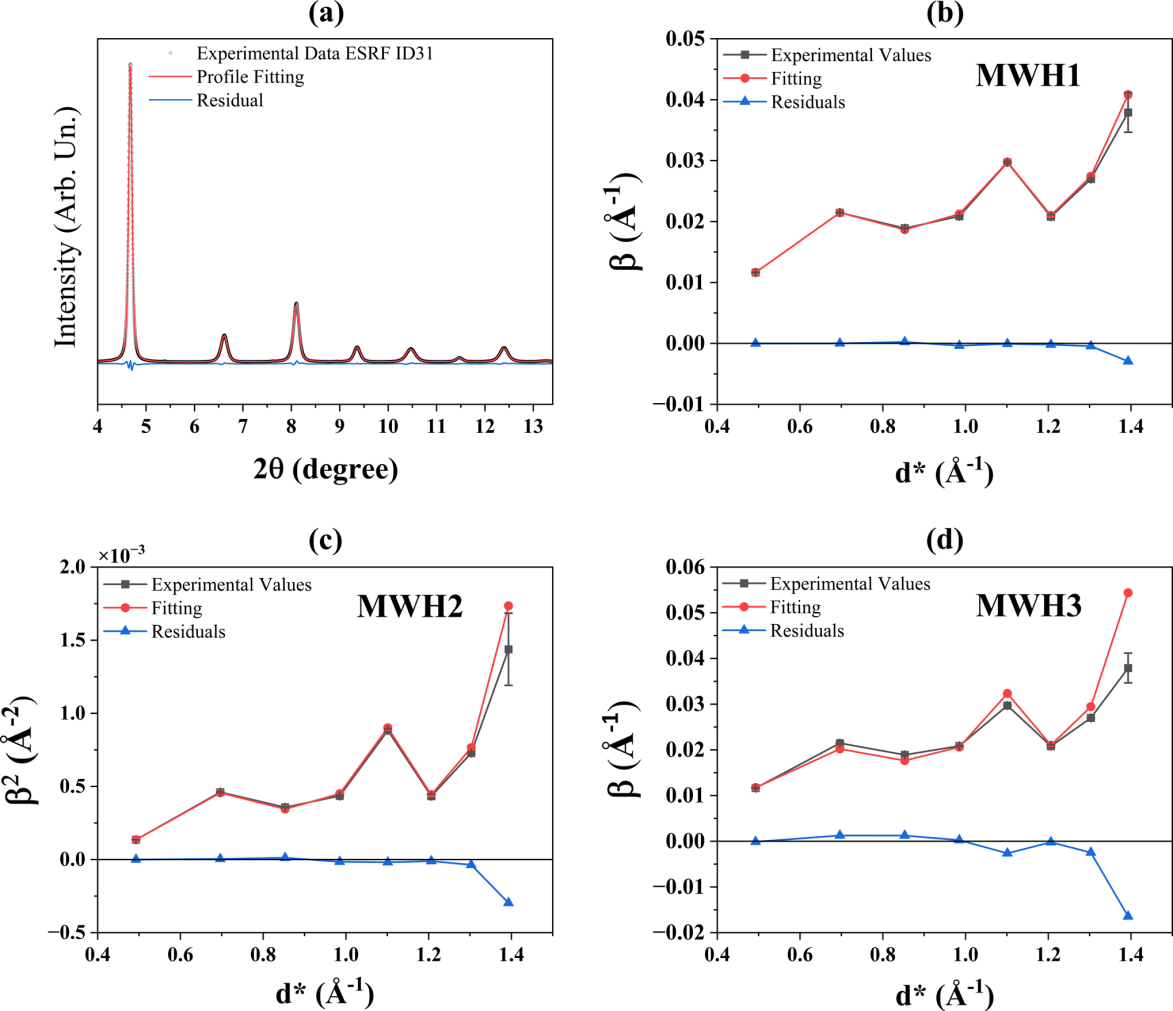
(*a*) Fitting using a Voigt function of the data obtained using setup (iii). Modified WH fittings using (*b*) equation (4), (*c*) equation (5) and (*d*) equation (6).

Fig. 7 ▸(*a*) shows again the modelling of the data collected on the 64 h-ball-milled FeCr powder with setup (iii) (ESRF beamline ID31). In this case, the broadening of the Voigtian profiles for CS and MS effects has been constrained to the MWH1 model of equation (4), with the result shown in Fig. 7 ▸(*b*). Similar modelling has been carried out using MWH2 [equation (5)] and MWH3 [equation (6)], shown in Figs. 7 ▸(*c*) and 7 ▸(*d*), respectively. All plots for the other measurement setups are reported in Supplementary Note 7. The two main parameters obtained from this analysis are 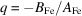 [equation (3)], which expresses the anisotropy of the MS, and 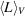, the effective domain size. The values are reported in Table 4 ▸, with the statistical indices (*R*_wp_ and GoF) of the *TOPAS* modelling.

The model that obtained values closest to WPPM is MWH3, with an average 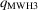 = 1.86 (2) and 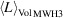 = 10.7 (5) nm, comparable to the WPPM average of 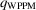
 = 1.92 (5) and 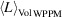
 = 13.9 (1) nm [value of 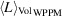 calculated from equation (15) of Scardi (2020 ▸)]. The result is confirmed with all the datasets of this study, collected in the different configurations. The WH method, and MWH3 in particular, although limited in the quantitative information that can be obtained, is confirmed to be simple and adequate for data comparison and screening. Also, for this analysis, FeCr powder has proven to be suitable for testing and benchmarking. Furthermore, 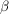 values such as those reported in Table S4 and Table 4 ▸ can be used to compare LPA models and results obtained with different experimental setups.

## 5. Conclusions

This study presents a comprehensive analysis of a ball-milled Fe–1.8%Cr steel powder, proposed as a reference material to test and compare powder diffraction instrumentation and data-modelling procedures. A line profile analysis carried out according to the whole powder pattern modelling approach assuming a lognormal distribution of equiaxed domains indicates an average size of 11.2 (3) nm, with a standard deviation of 4.7 (3) nm. The strong work hardening of the powder is demonstrated by the high dislocation density and yield-strain estimate obtained from the Warren plot, which reaches the highest values characteristic of highly deformed nanocrystalline steels. The powder, stable and readily available in large volumes, proves to be a suitable material for comparing different measurement geometries, including those of laboratory diffractometers and synchrotron beamlines for powder diffraction. The powder patterns are suitable for testing line profile analysis methods, as well as for deriving and studying the pair distribution function for a nanocrystalline material with pronounced microstructural effects.

## Supplementary Material

Supporting information. DOI: 10.1107/S1600576725006946/uu5017sup1.pdf

## Figures and Tables

**Table 1:** Refined parameters obtained by WPPM with different diffraction setups: ρ is the average dislocation density, *R*_e_ is the effective outer cut-off radius, *f*_E_ is the fraction of edge dislocations and *q* = −*B*/*A*, as in equation (3) Other parameters are described in the main text.

Sample	*R*_wp_ (%)	GoF	Lattice parameter (nm)	Average diameter (nm)	Standard deviation (nm)	DW (Å^2^)	ρ (nm^−2^)	*R*_e_ (nm)	*f*_E_	*q*
Co *K*α [setup (i)]	2.333	1.019	0.286948 (6)	12.1 (7)	4.2 (5)	0.12 (2)	0.041 (2)	6.4 (5)	0.62 (4)	1.88 (6)
Cu *K*α [setup (ii)]	12.57	1.082	0.28685 (3)	11 (2)	4 (1)	NaN	0.030 (2)	10 (1)	0.62 (4)	1.89 (5)
ID31† [setup (iii)]	1.56	6.15	0.28710 (1)	10.5 (2)	5.0 (2)	0.352 (2)	0.0290 (2)	10.3 (1)	0.56 (3)	1.962 (4)
ID31‡ [setup (iii)]	1.73	0.559	0.289 (2)	10 (2)	5 (2)	0.328 (3)	0.0284 (9)	8.3 (6)	0.55 (1)	1.99 (1)
ID22 [setup (iv)]	2.679	11.48	0.287226 (3)	9.5 (6)	4.8 (5)	0.347 (4)	0.0269 (6)	10.9 (6)	0.581 (9)	1.94 (1)

† SDD of 1500 mm.

‡ SDD of 300 mm.

**Table 2:** Young’s modulus, yield strain, yield stress and modulus of resilience estimated for each [*hkl*] direction

*h*	*k*	*l*	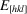 (GPa)	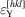	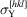 (GPa)	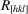 (MJ m^−3^)
2	0	0	135	0.0135	1.82	12.3
1	1	0	223	0.0099	2.21	10.9
2	2	2	286	0.0083	2.38	9.9
3	1	0	157	0.0123	1.94	12.0

**Table 3:** Structural and microstructural parameters obtained via PDF analysis

Measurement	*R*_wp_ (%)	Lattice parameter (nm)	Average diameter (nm)	Standard deviation (nm)	DW (Å^2^)	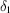	ɛ (× 10^−3^)
ID31† [setup (iii)]	9.22	0.28665 (5)	7.0 (0.8)	5.6 (3)	0.366 (2)	1.314 (2)	5.8 (4)
ID22 [setup (iv)]	9.3	0.287189 (2)	9 (2)	7.8 (7)	0.272 (1)	0.821 (8)	11.0 (1)

† SDD of 300 mm.

**Table 4:** Parameters for the MWH fitting

Setup/model	*R*_wp_ (%)	GoF	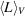 (nm)	*q*
(i)/MWH1	0.5128	0.679	70 (20)	1.8 (1)
(i)/MWH2	1.09	0.72	20 (4)	1.8 (2)
(i)/MWH3	1.0047	1.330	11.7 (5)	1.8 (2)
(ii)/MWH1	0.58	0.32	45 (4)	1.97 (5)
(ii)/MWH2	1.54	0.428	17 (1)	1.95 (6)
(ii)/MWH3	3.855	2.136	10.9 (7)	1.8 (3)
(iii)/MWH1	0.714	2.63	48 (4)	2.05 (5)
(iii)/MWH2	1.93	3.55	18.0 (9)	2.03 (7)
(iii)/MWH3	4.25	15.67	11.4 (6)	1.92 (2)
(iv)/MWH1	1.621	1.278	66 (9)	1.97 (6)
(iv)/MWH2	3.823	1.507	21 (2)	1.97 (6)
(iv)/MWH3	6.97	5.499	11.0 (5)	1.9 (2)

## Footnotes

1 If not available from commercial distributors, sample aliquots may be requested from the authors while supplies last. Even after the abundant available batch has been exhausted, it will be possible to produce a new one, so that the material can be used as a reference for a large community of powder diffraction users.

2 The equation reported by Lucks *et al.* (2001 ▸) assumes that the FWHM dynamic and static components are summed: .
