# Supplementary material for: A reference material for X-ray diffraction line profile analysis
Source: J Appl Crystallogr. 2025 Sep 18;58(Pt 5):1764–77. doi: 10.1107/S1600576725006946 (PMC12502865; doi:10.1107/S1600576725006946)
Supplement: Supplementary file 1 [file j-58-01764-sup1.pdf]

JOURNAL OF
APPLIED
CRYSTALLOGRAPHY

Volume 58 (2025)

Supporting information for article:

A reference material for XRD line profile analysis

P. Scardi, M. D'Incau, M. A. Malagutti, M. W. Terban, B. Hinrichsen and A. N. Fitch

S1. Supplementary Note 1: Details of ID31 beamline

Synchrotron X-ray diffraction and total scattering [1], [2] measurements were performed at beamline ID31 at the European Synchrotron Radiation Facility (ESRF). The sample powders were loaded into cylindrical slots (approx. 1 mm thickness) held between polyimide windows in a high-throughput sample holder. Each sample was measured in transmission geometry with an incident X-ray energy of 75.60 keV (λ = 0.1640 Å). Measured intensities were collected using a Pilatus CdTe 2M detector (1679 × 1475 pixels, 172 × 172 μm^2 each) positioned with the incident beam in the corner of the detector. The sample-to-detector distance was approximately 1.5 m for the high-resolution measurements and 0.3 m for total scattering. Multiple measurements of the empty well with polyimide windows were measured, and summed together for improved statistics, for the background subtraction. NIST SRM 660b (LaB_6) was used for geometry. Integration was performed with the software pyFAI including flat-field, geometry, solid-angle, and polarization corrections. Invalid pixels were masked, and the sigma clipping integration algorithm was used to auto-mask further azimuthal outliers. The summed background intensities were scaled respectively to each of the reference and sample measurements and subtracted. Rietveld refinements were performed using TOPAS v7 [3] to fit the structure with the reference lattice parameter fixed to the measurement to determine the instrumental profile contribution and correct for offset errors including parallax [4]. PDF data were processed using PDFgetX3 [5]–[7] with a Qmax of $30 Å^{-1}$.

S2. Supplementary Note 2: Details of the ICP-OES characterization

Approximately 0.2 g of powder is weighed into a 50 mL volumetric flask that has been previously cleaned with concentrated nitric acid. Then, 9 mL of ultrapure hydrochloric acid and 3 mL of nitric acid are added, heating until complete dissolution is achieved. After cooling, the volume is brought to the mark with pure water. The following products were using for the preparation: Ultrapure nitric acid 70% (Chem Lab), ultrapure hydrochloric acid 36% (Aldrich), multielement standard for ICP solution I (Fluka), and pure water produced by reverse osmosis (conductivity <0.1 µS) are used. The analysis of major elements is performed using ICP standards, diluted in 1% nitric acid, with a maximum concentration of 5 ppm.

S3. Supplementary Note 3: LaB_6 fitting

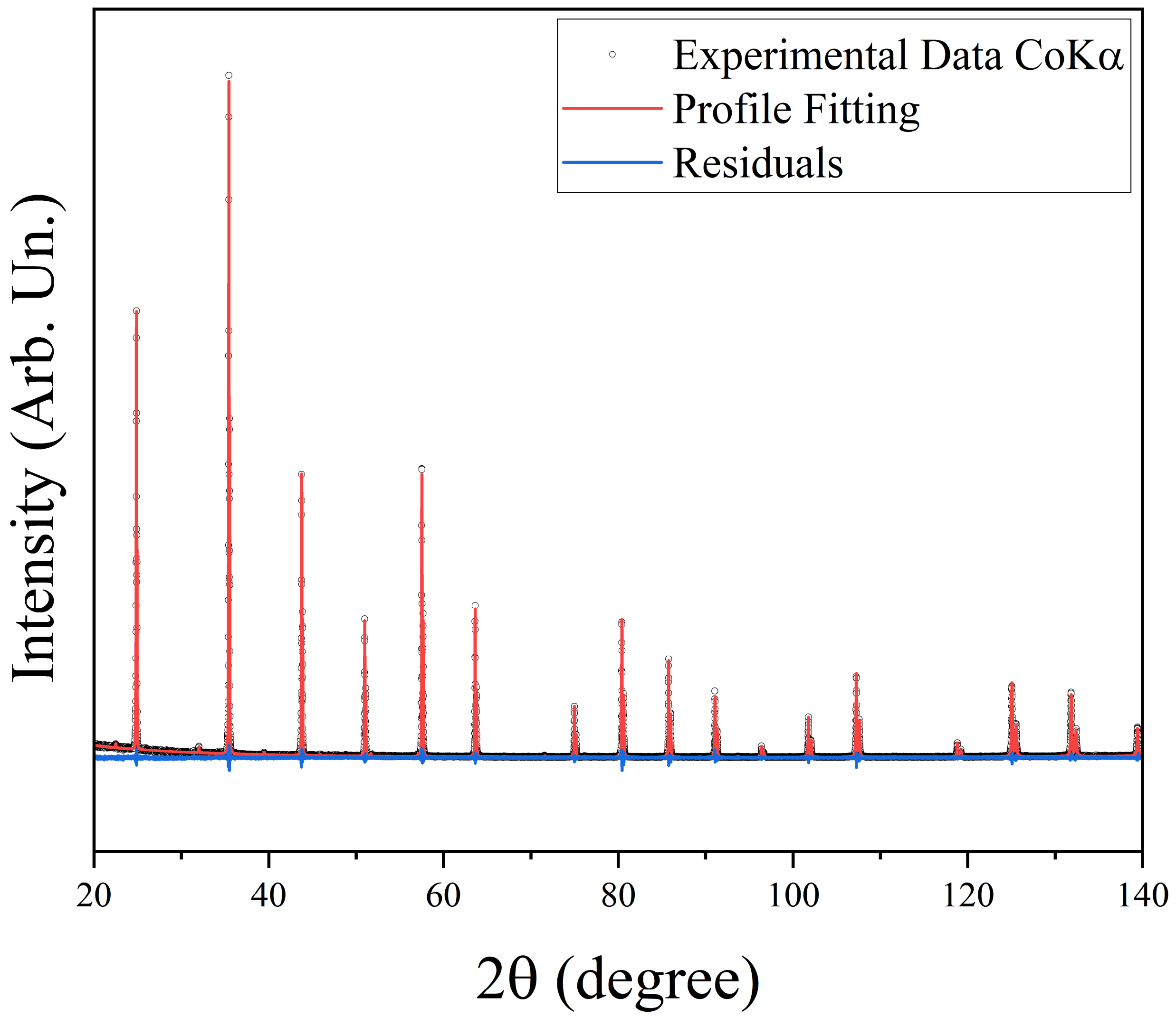

Figure S1 LaB6 XRD fitting used for obtaining the IRF for the CoKα radiation [setup (i)].

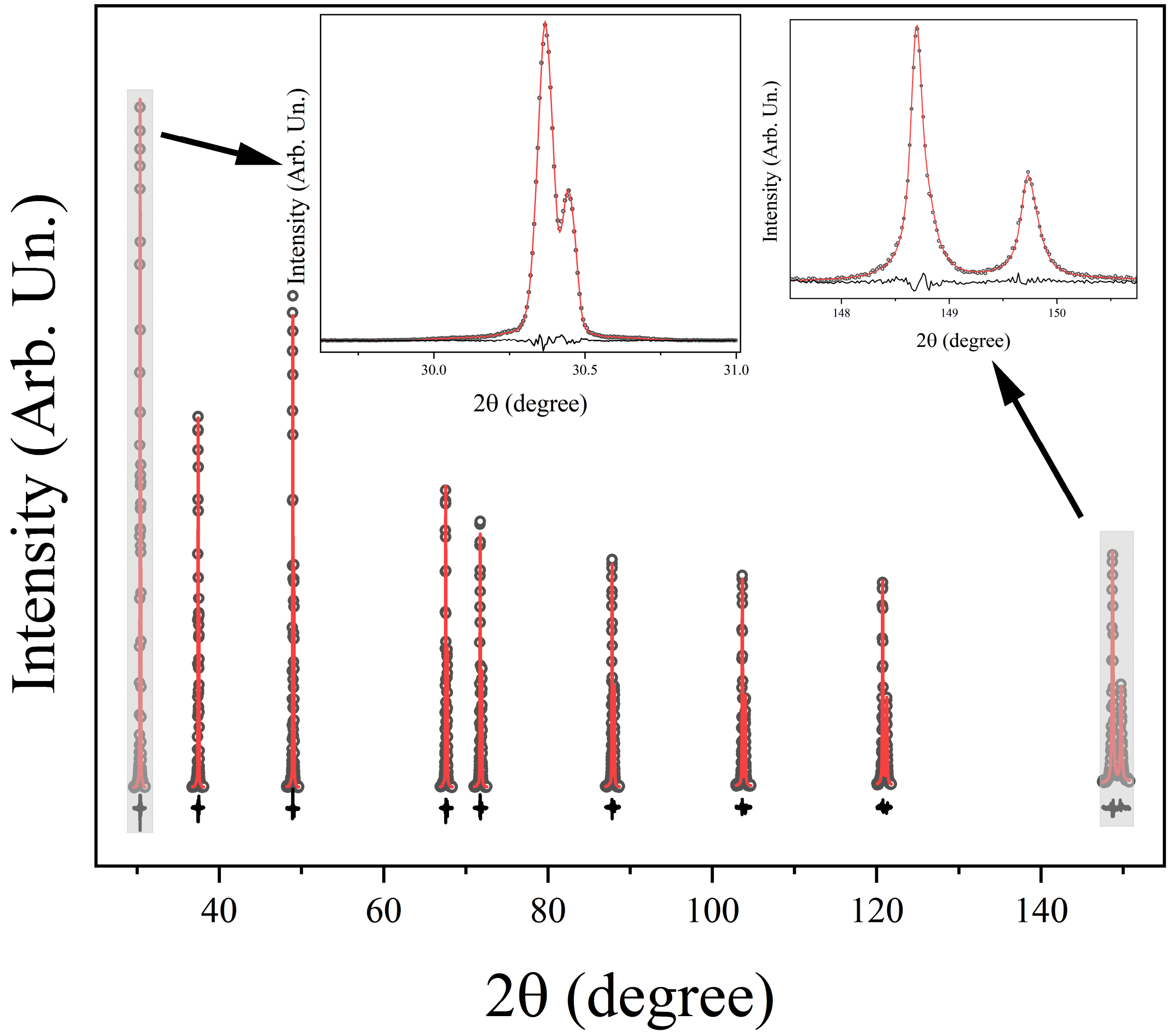

Figure S2 LaB6 XRD fitting used for obtaining the IRF for the CuKα radiation [setup (ii)].

S4. Supplementary Note 4: Correlation coefficients

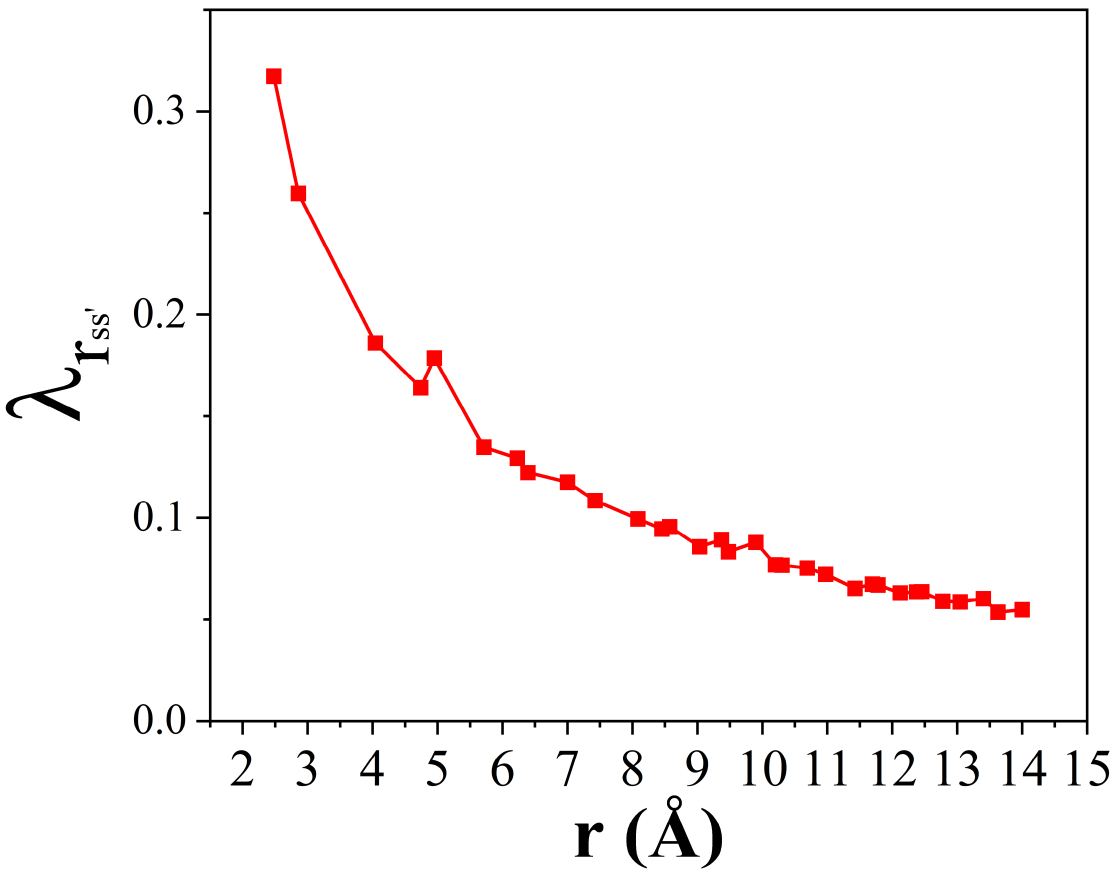

Figure S3 Correlation coefficients obtained via Fe nanosphere sumulations.

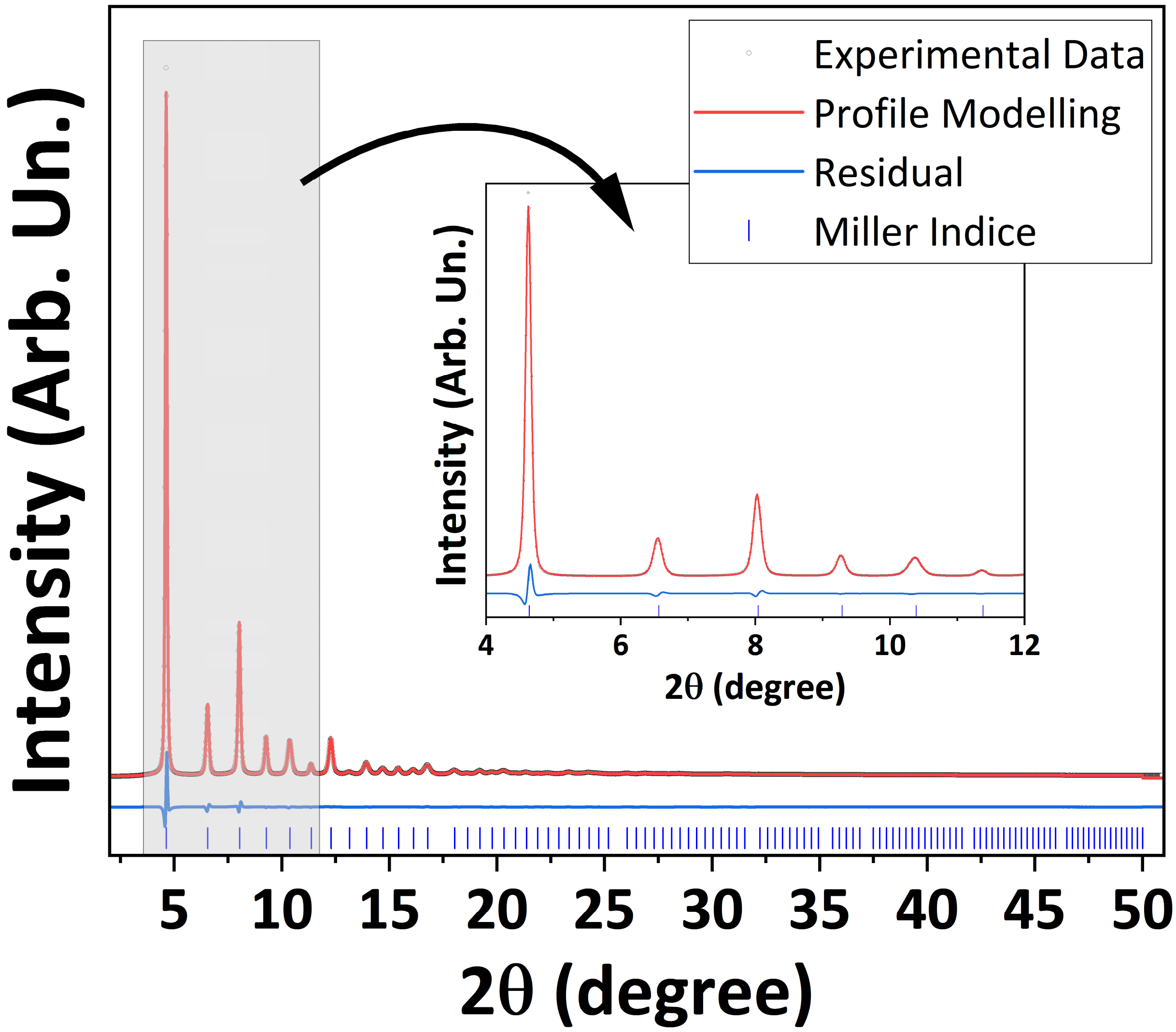

Figure S4 WPPM fitting of the ID31 data (setup iii – SDD of 300 mm). Dots represent the experimental data, red line the profile modelling, blue line the residual, and the thick markers represent the Miller indices.

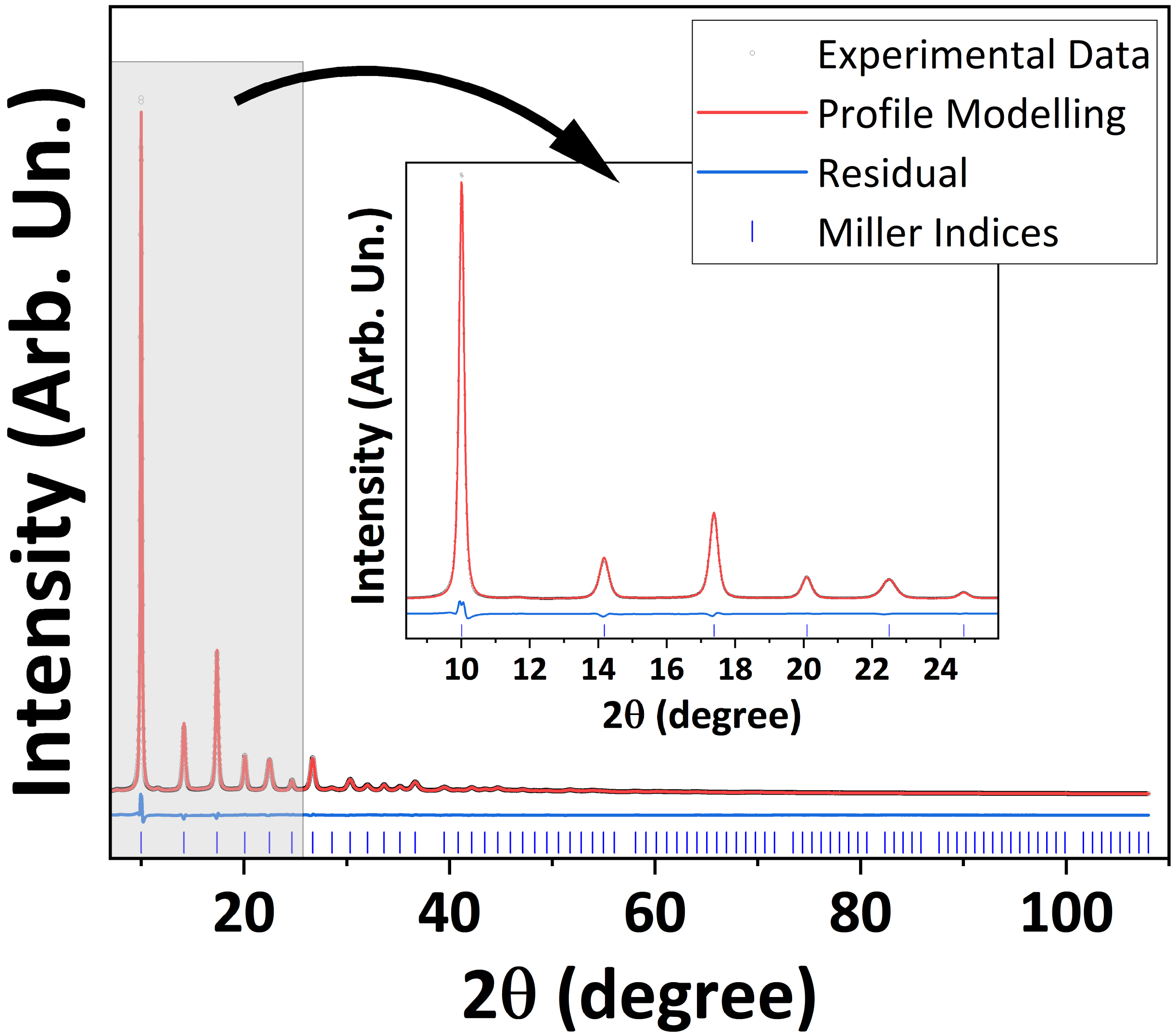

Figure S5 WPPM fitting of the ID22 data (setup iv). Dots represent the experimental data, red line the profile modelling, blue line the residual, and the thick markers represent the Miller indices.

S5. Supplementary Note 5: ICP-OES and EDXS analyses

Table S1 Atomic percentage of each element in the sample measured via EDXS.

Milling Time	Fe %	Cr%	Al%	Si%	Cu%	Ni%
**0h**	97(3)	1.85(9)	0.72(5)	0.15(3)	0.03(2)	0.04(2)
**8h**	97(3)	1.95(9)	0.59(5)	0.13(2)	0.08(3)	0.12(4)
**64h**	97(3)	1.93(9)	0.76(6)	0.13(2)	0.05(2)	0.14(4)

Table S2 ICP-OES analysis.

		ppm Cr 205.552		ppm Mn 257.611		ppm Ni 231.604		ppm Cu 324.754	
	**Grams of material**	**solution**	**solid**	**solution**	**solid**	**solution**	**solid**	**solution**	**solid**
**0H**	0.2941	69	11731	0.48	82	1.35	230	0.58	99
**64H**	0.2793	77	13784	2.74	491	5.5	985	0.99	177

The analysis of Cr is semi-quantitative as its concentration significantly exceeds the maximum calibration limit (6 ppm). The values for Mn, Ni, and Cu are accurate and have been cross verified on other lines.

S6. Supplementary Note 6: PDF fitting of ESRF ID22 and ID31 beamline data

Three parameters are required in order to describe the instrumental/measurement aberrations in the PDF.

I) The first one involves the limiting resolution, *i.e.*, the q step of the reciprocal data, requiring that a Gaussian enveloping function in the form $\exp[-q_{damp}r^2/2]$ must multiply G(r), where q_{damp} must be empirically estimated using the LaB_6 standard, that presumably has no size/strain effects. This expression can be extended to a pseudo-voigt (PV) profile in the form:

$$Damp = (1-\eta)\exp\left(-\frac{1}{2}\,r^2\left(\frac{q_{damp}}{2\sqrt{2\,\mathrm{Ln}(2)}}\right)^2\right) + \eta\exp\left(-\frac{1}{2}q_{damp}\,r\right)$$

II) The second contribution comes from the high noise at higher Q values in the reciprocal space, that when is transformed to real space via Fourier transformation, adds a broadening to the PDF peaks with the following variance assuming that the PDF peaks are gaussians:

$$\sigma^2_{broad} = \delta^2_{broad} r^2_{ss'}$$

In the table below the values obtained for the PDFgui and TOPAS softwares are shown.

Table S3 Parameters for the instrumental contribution to the PDF profile.

Ref. No.	Beamline	Refinement Software	Range in r (Å)	q_{damp}	Lorentzian Mixing Parameter η	δ_{broad}
**1**	ID22	PDFgui	1.4–75	0.002545	–	0.0029
**2**	ID31	PDFgui	1.4–75	0.01174	–	0.014482
**3**	ID22	TOPAS	1–100	0.000899	0.432678	0.001512
**4**	ID31	TOPAS	1–110	0.029108	0	0.02737

In addition, data truncation effects, expressed by the limiting q_{max} in the reciprocal space representation, introduces termination ripples in the Fourier transform. These are often modelled by convolving the PDF with a $\mathrm{Sinc}(q_{max}r)$ function.

S7. Supplementary Note 7: Modified Williamson-Hall plots

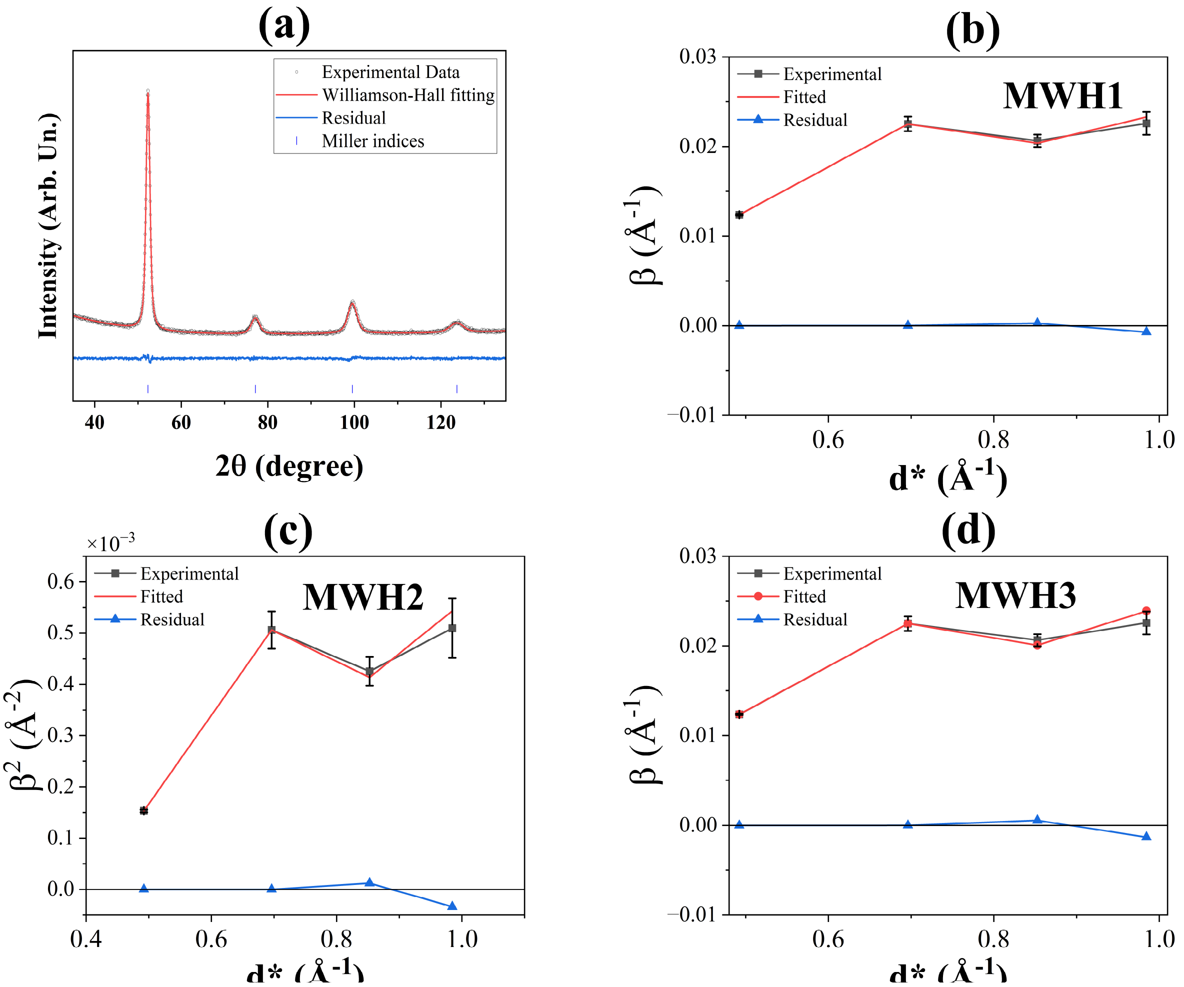

Figure S6 (a) Double-Voigt fitting of the Fe1.8Cr sample ball-milled at 64 h and measured using CoKα radiation (setup(i)). MWH fitting of the integral breadth vs d* using (b) MWH1, (c) MWH2, and (d) MWH3 models.

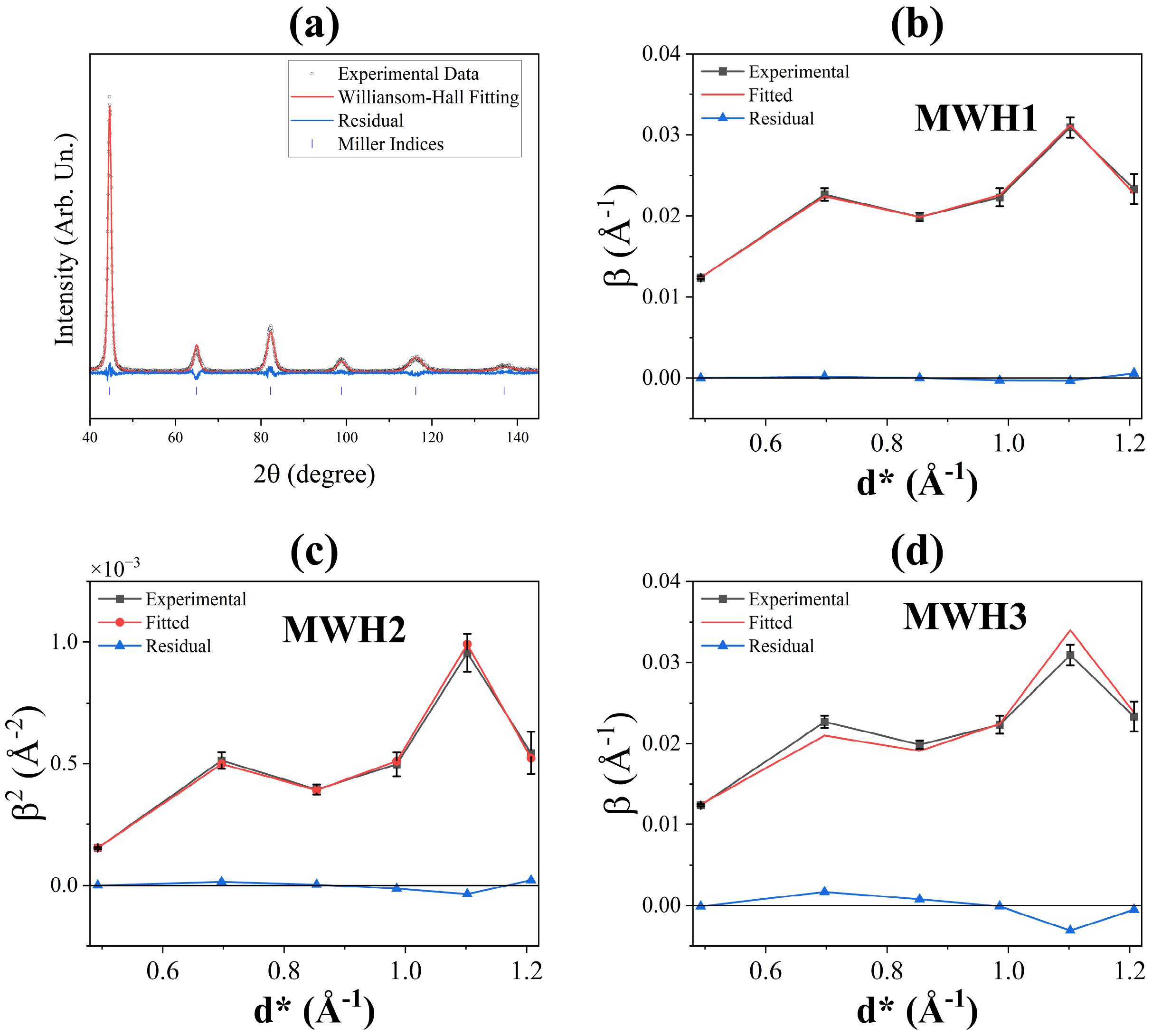

Figure S7 (a) Double-Voigt fitting of the Fe1.8Cr sample ball-milled at 64 h and measured using CuKα radiation (setup(ii)). MWH fitting of the integral breadth vs d* using (b) MWH1, (c) MWH2, and (d) MWH3 models.

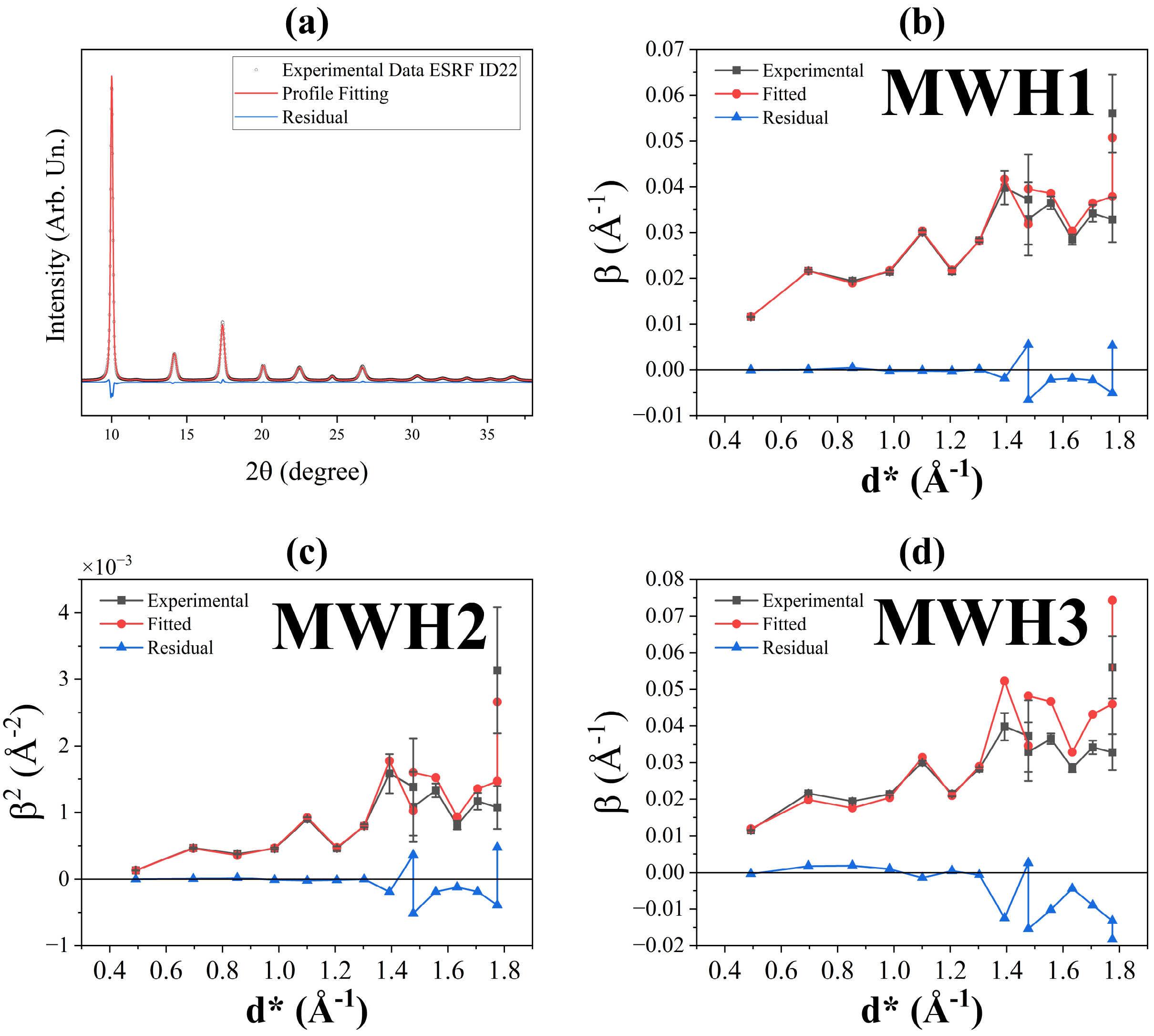

Figure S8 (a) Double-Voigt fitting of the Fe1.8Cr sample ball-milled at 64 h and measured using ESRF ID22 (setup(v)). MWH fitting of the integral breadth vs d* using (b) MWH1, (c) MWH2, and (d) MWH3 models.

Table S4 Table of integral breadth versus d* for all the measurements performed on the $Fe_{1.8}Cr$ sample ball-milled at 64 h.

**Measurement Setup**	Miller indices	$d^*(Å^{-1})$	$\beta(Å^{-1})$	$\beta(Å^{-1})$ **error**
**(i)**	(110)	0.492270	0.0123691	9.91914e-05
	(200)	0.696175	0.02249762	0.0008117994
	(211)	0.852637	0.02062685	0.0006806809
	(220)	0.984540	0.02257294	0.001282618

**(ii)**	(110)	0.492885	0.01235994	0.0001517698
	(200)	0.697044	0.0222316	0.0007847683
	(211)	0.853702	0.01938339	0.0004928153
	(220)	0.985770	0.01974166	0.0009819346
	(222)	1.207316	0.01844522	0.001989843
	(310)	1.102124	0.02688631	0.001004674
**(iii) – SDD 1500mm**	(110)	0.492511	0.01164239	1.785793e-05
	(200)	0.696516	0.02146793	9.17628e-05
	(211)	0.853054	0.0189067	5.659519e-05
	(220)	0.985022	0.02086084	0.0001334871
	(222)	1.206400	0.02080932	0.0003350363
	(310)	1.101288	0.02970855	0.0001699629
	(321)	1.303061	0.0269932	0.0001589453
	(400)	1.393031	0.03791512	0.003256723
**(iv)**	(110)	0.49235	0.01156	6.6733E-5
	(200)	0.69628	0.02159	2.59886E-4
	(211)	0.85277	0.01944	1.65833E-4
	(220)	0.98469	0.02133	3.29303E-4
	(222)	1.206	0.02147	6.89415E-4
	(310)	1.10092	0.02999	4.07878E-4
	(321)	1.30263	0.02828	3.87652E-4
	(330)	1.47704	0.03593	0.00901
	(332)	1.63293	0.02846	0.00112
	(400)	1.39257	0.03982	0.00365
	(411)	1.47704	0.03301	0.00766
	(420)	1.55694	0.0365	0.0014
	(422)	1.70554	0.03419	0.00185
	(431)	1.77518	0.03269	0.00501
	(510)	1.77518	0.05543	0.00839

S8. References

[1] M. W. Terban and S. J. L. Billinge, “Structural Analysis of Molecular Materials Using the Pair Distribution Function,” *Chem. Rev.*, vol. 122, no. 1, pp. 1208–1272, 2022.

[2] T. Egami and S. J. L. Billinge, *Underneath the Bragg peaks: structural analysis of complex materials*. 2003.

[3] A. A. Coelho, "TOPAS and TOPAS-Academic: An optimization program integrating computer algebra and crystallographic objects written in C++: An," *J. Appl. Crystallogr.*, vol. 51, no. 1, pp. 210–218, Feb. 2018.

[4] F. Marlton, O. Ivashko, M. V. Zimmerman, O. Gutowski, A. C. Dippel, and M. R. V. Jørgensen, "A simple correction for the parallax effect in X-ray pair distribution function measurements," *J. Appl. Crystallogr.*, vol. 52, pp. 1072–1076, 2019.

[5] P. F. Peterson, E. S. Božin, T. Proffen, and S. J. L. Billinge, "Improved measures of quality for the atomic pair distribution function," *J. Appl. Crystallogr.*, vol. 36, no. 1, pp. 53–64, 2003.

[6] S. J. L. Billinge and C. L. Farrow, "Towards a robust ad hoc data correction approach that yields reliable atomic pair distribution functions from powder diffraction data," *J. Phys. Condens. Matter*, vol. 25, no. 45, 2013.

[7] P. Juhás, T. Davis, C. L. Farrow, and S. J. L. Billinge, "PDFgetX3 : a rapid and highly automatable program for processing powder diffraction data into total scattering pair distribution functions," *J. Appl. Crystallogr.*, vol. 46, no. 2, pp. 560–566, Apr. 2013.
